# A Synergistic Mindsets Intervention Protects Adolescents from Social Stress

**DOI:** 10.21203/rs.3.rs-551170/v1

**Published:** 2021-05-28

**Authors:** David Yeager, Christopher Bryan, James Gross, Danielle Krettek, Pedro Santos, Jared Murray, Hannah Graveling, Meghann Johnson, Jeremy Jamieson

**Affiliations:** University of Texas at Austin; University of Texas at Austin; Stanford University; Google Empathy Lab; University of Texas at Austin; University of Texas, Austin; University of Rochester; University of Texas at Austin; University of Rochester

**Keywords:** adolescent health, anxiety, depression, social stress

## Abstract

Social stress poses a major threat to adolescent health via its effects on internalizing symptoms, such as anxiety and depression. Available interventions to help adolescents improve their stress responses, however, have not been effective in rigorous evaluation studies, or they have been difficult to administer widely. Here we show that replicable improvements in adolescent stress responses can be achieved with a short (~30-minute), scalable synergistic mindsets intervention. This intervention, which is a self-administered online training module, targets both growth mindsets (the idea that people’s intelligence can be developed in response to challenge) and stress-can-be-enhancing mindsets (the idea that people’s stress responses can be fuel for optimal performance). Its goal is to promote positive engagement with stressful events (e.g., learning from failure on a quiz or a conflict with a peer) and to encourage adolescents to use their responses to stressful events and even their bodily symptoms (e.g. racing heart, sweaty palms, butterflies in their stomach) to their advantage. In five double-blind, randomized, controlled trials (total N = 4,091 adolescents), the new synergistic mindsets intervention improved stress-related cognitions (Studies 1-2), cardiovascular reactivity (Study 3), daily internalizing symptoms and cortisol levels (Study 4), and generalized anxiety symptoms during the 2020 COVID-19 lockdowns (Study 5). Effects on downstream outcomes (in Studies 3-5) were stronger among individuals who, at baseline, held the two negative mindsets targeted by the intervention, providing evidence for the proposed mechanisms. Confidence in this conclusion comes from a conservative, Bayesian machine-learning method for detecting heterogeneity.

Adolescents today show record levels of stress-related internalizing symptoms ^1-7^. This has prompted public health experts to call for urgent action to mitigate the coming “mental health pandemic^8^” by understanding and addressing adolescent stress ^9-11^.

Conventional thinking suggests that stress is bad and should be avoided ^12-14^. This may sometimes be misguided, however, because stress is a normal and even defining feature of adolescence ^15^. For example, adolescents must acquire a wide and varied array of complicated social and intellectual skills as they transition to adult social roles and join the labor market. This process is inherently stressful, but it is also essential to the task of becoming an adult^15^. Furthermore, if adolescents simply disengaged from their stressors, it could put them at a serious disadvantage in the future. Technology has displaced many low-skill jobs and created more highly technical ones^16^. This compels adolescents to complete more advanced coursework in math and science ^17,18^. Such cognitively demanding coursework can evoke higher levels of stress^19^, even though it must be taken to compete for higher-wage positions^20^. The COVID-19 pandemic has accelerated recent economic trends while adding the intense and persistent stressors of social isolation and uncertainty about the future^1-3^. If adolescents wish to succeed in the labor market and overcome threats to personal and global survival, they must find a way to embrace and overcome the stressful demands of social and intellectual development.

To address this pressing challenge, affective scientists have increasingly advocated for a stress *optimization* approach, which refers to helping adolescents positively engage with common and potentially-beneficial social and academic stressors, rather than avoiding stress altogether ^21,22^. To date, however, the search for adolescent stress optimization interventions has been largely unsuccessful. Indeed, meta-analyses have reported mostly negligible protection against internalizing responses to stressors in universal adolescent populations ^23-25^.

Here we show that it is possible to achieve stress optimization by targeting adolescents’ beliefs, or *mindsets*, about their stressful experiences. We demonstrate that a short (~30-minutes), scalable intervention that could, in principle, be administered to national populations of adolescents^26^, optimized stress responses at each step on the path from stressful demands to internalizing symptoms: from cognitive to cardiovascular to neuroendocrine responses (see Fig 1A).

The intervention evaluated here, called the *synergistic mindsets* intervention, targeted two different mindsets. The first is an academic *growth mindset*^27^. This is the belief that intellectual ability is not fixed but can be developed with effort, effective strategies, and support from others ^26,28,29^. Adolescents who receive this message are taught that failures are potentially helpful (because they could be opportunities for learning and growing abilities) and controllable (because you can take steps to grow your abilities and avoid future failures). The second is the *stress-can-be-enhancing mindset* ^14,21^. Adolescents were taught that their mind’s and body’s responses to stressful experiences (e.g., feelings of worry, sweaty palms, racing heart, deeper breathing) can be positive (because you are mobilizing energy and delivering oxygenated blood to the brain and tissues) and can be controlled (because you are harnessing the energy from stress and using it to optimize performance).

In the intervention, these two mindsets were not presented to adolescents as separate ideas, but were intertwined. The growth mindset messaging encouraged adolescents to embrace, and not avoid, the difficulty and challenge in a situation. When engagement inevitably feels stressful, the stress-can-be-enhancing mindset led adolescents to “lean into” that stress response and use it to pursue valued goals. These combined messages led adolescents to view stressors as things that could be overcome, rather than as things that made them feel overwhelmed.

This synergistic approach goes beyond past studies that did not appreciate the complementarity between two different kinds of mindsets: mindsets about negative *events* (e.g., academic failures, the focus of a growth mindset message) and mindsets about stress *responses* (e.g., feelings of worry, butterflies in your stomach, the focus of a stress-can-be-enhancing message). Past intervention studies have targeted one or the other of the two mindsets ^14,26^, but only a few, isolated studies to date have found meaningful changes in adolescents’ stress responses and some have found no effects ^30^.

We argue that the two mindsets—about *events* and *responses*—needed to be integrated to reliably optimize stress responses in real-world settings (see Fig. 1A and 1B). For example, if an adolescent believes that stressful failures can help fuel learning (an event-focused growth mindset of intelligence), but also believes that their psychological or physiological stress responses are harmful and uncontrollable, leading them to feel “stressed about being stressed” ^21^ (a response-focused stress-is-debilitating mindset), they may still shy away from a stressful learning experience. Likewise, an adolescent who thinks that stress responses can be helpful and controlled (a response-focused stress-can-be-enhancing mindset) may not think to utilize their stress responses if they believed that failures are inherently undermining or harmful and cannot be controlled anyway (an event-focused fixed mindset of intelligence). By targeting both mindsets simultaneously, the synergistic mindsets intervention can convey the positive message that both stressful events *and* stress responses can be helpful and controlled.

This intervention overcomes the primary limitation of the popular “reappraisal” approach to promoting well-being. A person’s cognitive appraisal is the meaning that stressful situations can have for the individual ^31^, and it can shape their stress responses ^5,31-34^. In past laboratory experiments, leading people to reappraise stress as helpful and controllable (vs. unhelpful and uncontrollable) improved immediate cognitive, physiological, and behavioral stress responses ^5,32-34^. Nevertheless, appraisal-focused approaches suffer from the *transfer problem*: people usually fail to show effects of a reappraisal treatment on untrained stimuli—which are virtually all of the stimuli or events the individual later encounters ^35-38^. For instance, an adolescent might be convinced to appraise their struggles on one important exam as helpful and controllable, but they may not make the same appraisal of other failures. The synergistic mindsets intervention solves the transfer problem by targeting cognitive processes that operate at a more general level than situation-specific appraisals: mindsets. Mindsets affect how people think about categories of situations (e.g., academic failures or negative emotions in general) ^14,39,40^, and therefore help people deductively make appraisals about the meanings of situations they have not been trained on. Thus, benefits of mindset interventions can transfer across situations, even novel ones (such as how to deal with the stress of an unexpected global pandemic).

## The Present Research

In a series of experiments we assessed the effects of the synergistic mindsets intervention in formal education settings (e.g., taking a timed quiz, transitioning to high school, being isolated from school peers) because a primary source of adolescents’ evaluative stress comes from navigating a volatile social world while also acquiring the academic skills that prepare them for adulthood ^41^. Our sample included mid-to-late adolescents who were on the cusp of transitioning to adult roles (i.e. in secondary school or in the first two years of post-secondary education). Adolescents completed the online intervention module on their own, usually as a classroom activity, without assistance from a trained professional and without discussing with instructors. Hence, the study procedures mirrored the routine conditions under which scale-up could occur.

**Figure 1:**
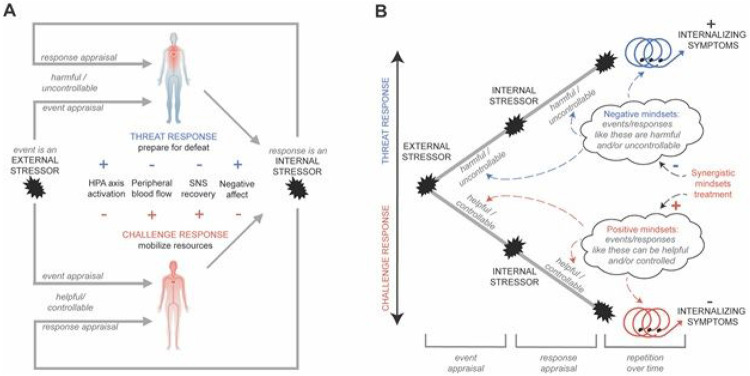
The model guiding the present study’s predictions. (A) In an acute situation, differences in appraisals lead to differences in challenge versus threat responses. (B) Mindsets lead to differences in appraisals and shape responses in acute situations and across situations over time. In the event appraisal stage, stressors are appraised as harmful/uncontrollable or more helpful/controllable, cultivating threat or challenge response tendencies, respectively. Then, at the response appraisal stage, when individuals actively engage with stressors, the meaning of their stress response is appraised as either distressing and non-functional (harmful/uncontrollable) or as a resource that helps one address situational demands (helpful/controllable), resulting in further threat or challenge type stress responses, respectively. As shown in Panel A, challenge and threat responses differentially activate stress axes in the brain. Although both elicit sympathetic-adrenal-medullary (SAM) activation, threat also stimulates the hypothalamic-pituitary-adrenal (HPA) axis, the end-product of which is the catabolic adrenal hormone cortisol, in anticipation of damage or social defeat. Challenge is characterized by increased peripheral blood flow (which is why it is depicted in red), and an agile response onset/offset: resources are mobilized rapidly, and individuals return to homeostasis quickly after stress offset. Threat, however, results in increased vascular resistance and less oxygenated blood flow to the periphery (which is why it is depicted in blue) as HPA activation tempers SAM effects and produces a more prolonged stress response than challenge due to the longer half-life of cortisol compared to anabolic hormones. Challenge and threat then have different consequences for motivation and affective responses. Whereas threat leads to avoidance motivation and negative affect, challenge elicits approach motivation and more positive affect relative to threat. As shown in (B) mindsets are situation-general beliefs about categories of events (e.g., academic stressors) and responses (e.g., feelings of worry). The mindsets shape appraisals at the event stage and next at the response stage. Thus, mindsets “count twice” toward the construction of affective responses. Downstream, if individuals respond with an optimized challenge type stress response, this increases the likelihood they will engage with and respond to future stressors more adaptively in a self-reinforcing, positive feedback cycle, the end result of which is buffering against internalizing symptoms (bottom right in panel B).

Broadly, we expected to reduce indicators of negative *threat-type* stress responses. The sequential organization of the five experiments follows the levels of threat-type responses in Fig. 1, from cognitive appraisals to cardiovascular and neuroendocrine responses to internalizing symptoms (see Table 1). Threat-type stress responses stem from the appraisal that a stressor is “bad for me” and uncontrollable, which leads to the conclusion that one cannot handle the stressor ^42^ (i.e. a threat appraisal). Threat appraisals then elicit a cascade of negative consequences that signal preparation for damage and defeat (see Fig. 1A and B) ^42,43^.

## Effects on Cognitive Appraisals

In two large, pre-registered experiments we examined effects of the intervention on cognitive appraisal processes that function upstream from the key outcomes examined later in Studies 3-5. Throughout, we used a Bayesian statistical analysis approach and focus on effect sizes and uncertainty intervals rather than null hypothesis significance testing. (The findings also met conventional standards for statistical significance testing; see Extended Data Table 2).

As expected, Study 1 found that the intervention reduced negative *event*-focused appraisals of an anticipated academic stressor (e.g., “*How likely would you be to think that the very hard assignment [in your most stressful class] is a negative threat to you?*”), ATE = −.112 *SD* [−.029, −.200] and *response*-focused appraisals (e.g., “*I think my body’s stress responses would hurt my performance*”), ATE = −.191 *SD* [−.082, −.302], thus demonstrating the first steps in Fig 1.

**Figure 2:**
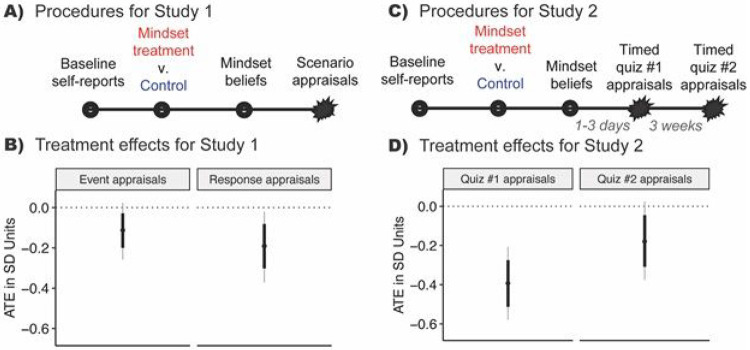
Two experiments (Study 1 N = 2,717; Study 2 N = 755) showed that the synergistic mindsets intervention reduced negative appraisals of an immediate, hypothetical stressor (A, B), and an acute naturalistic stressor up to 3 weeks post-intervention (C, D). Note: Starbursts represent stressor onset. Results estimated with the BCF algorithm. Thick lines represent the 10th to 90th %iles; gray lines represent the 2.5th to 97.5th %iles. ATE = average treatment effect. The appraisals for each study were coded so that higher values meant more negative appraisals, so negative treatment effects are consistent with a beneficial stress optimization effect. Effect sizes appear in the text.

Study 2 extended the effects of the intervention on appraisals of an experienced, acute stressor (Fig. 2). Immediately after a timed, challenging quiz in an undergraduate social science course, (which occurred 1-3 days post-intervention was not mentioned in the intervention content), treated participants made less-negative stress appraisals, ATE = −.392 *SD* [−.275, −.513], This effect persisted but was attenuated ~50%, at the 3-week follow-up, ATE = −.179 *SD* [−.045, −.308], Even so, this attenuated effect size was indistinguishable from the immediate appraisals in Study 1 and was sizeable considering there were no boosters or reminders of the content. Thus Study 2 showed that participants transferred the intervention message to new stimuli over time.

## Effects on Physiological Responses

**Figure 3:**
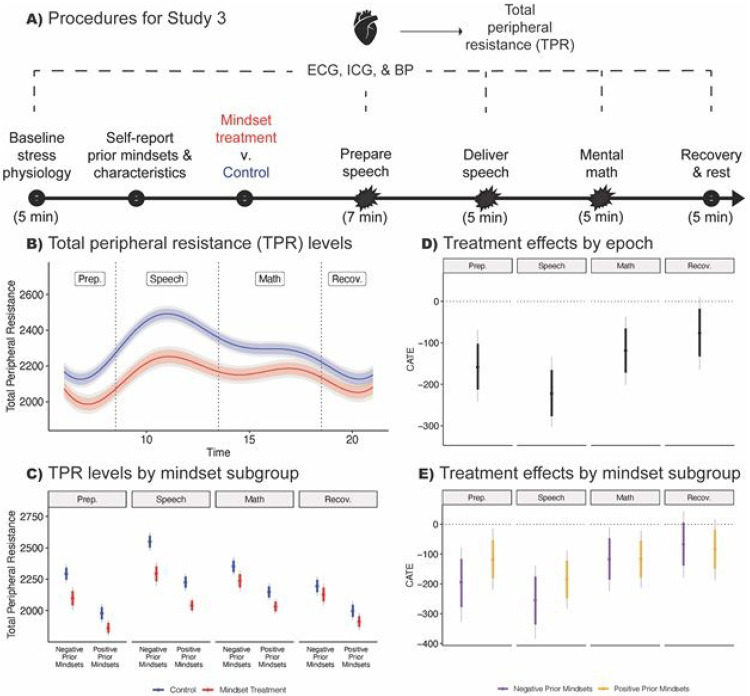
In Study 3 (N = 160), the synergistic mindsets intervention improved cardiovascular responses to the Trier Social Stress Test (TSST) overall (B,D) and especially for participants with negative prior mindsets during the most stressful epochs (C, E). Note: ATE = Average treatment effect. CATE = Conditional average treatment effect. TPR = total peripheral resistance (in dyne-sec x cm5). ICG = Impedance cardiography. BP = Blood pressure. ECG = Electrocardiography. Time indicates elapsed, cumulative physiological recording. TSST = Trier Social Stress Test. Starbursts indicate TSST epochs that presented acute demands (i.e., the stressful epochs). Baseline measurements were taken prior to the stress induction and random assignment to condition, preparation measurements were taken after intervention materials when participants planned their speech, speech delivery and mental math measurements were taken during the speech and math tasks, respectively, and finally measurements during a recovery period, where evaluative pressure (i.e., stress) was removed, tracked recovery to baseline. Data reported here control for baseline values. Thick / colored lines represent the 10th to 90th %iles; gray lines represent the 2.5th to 97.5th %iles. ATEs and 10th to 90th%iles for Preparation = −158.307 dyne-sec x cm5 [−212.458, −102.174], Speech = −221.976 [−276.696, −165.540], Math = −118.571 [−171.965, −36.903], Recovery = −76.245 [−132.624, −17.962]. The prior mindset subgroups used to display treatment effects in (C) and (E) were generated by implementing a hands-off Bayesian decision-making algorithm that maximized the differences among the mindset groups in terms of the outcome, without using information on the magnitudes of the treatment effects (see online supplemental material).

Study 3 used a well-validated, standardized acute stress induction paradigm (the Trier Social Stress Test, or the TSST ^44^; see also ^45^) to test for effects of the intervention on cardiovascular responses. Fig. 3A below depicts the five TSST epochs during which electrocardiography (ECG), impedance cardiography (ICG), and blood pressure (BP) signals were monitored to assess stress responses, with the speech epoch expected to elicit the most distress. The focal outcome was Total Peripheral Resistance (TPR), a measure of overall resistance of blood flow throughout the circulatory system, which is a positive cardiovascular indicator of threat-type stress responses (see Fig. 1A) ^43,46^. Therefore we expected the intervention to reduce TPR levels.

### Average effects.

Control group participants exhibited an increase in TPR from the preparatory to active epochs (Fig. 3B). The spike in TPR, an indicator of negative threat-type stress responses, was most pronounced during the demanding impromptu speech. This is consistent with the literature that self-relevant public speaking in front of non-supportive evaluators is a potent negative stressor ^47^.

In the treatment condition, the spike in TPR was blunted during the speech, and treated adolescents recovered to baseline more quickly after stress offset (Fig. 3B). At every epoch of the TSST, and especially during the most-stressful speech epoch, there was a meaningful treatment effect. The expected TPR for the mindset treatment group was lower than the control group and the conditional average treatment effect (CATE) was less than zero (see Fig. 3C). Analyses of other cardiovascular indicators of threat versus challenge-type stress responses (stroke volume during active epochs, and pre-ejection period during the final, recovery epoch, after stress offset) yielded treatment effects as well (Extended Data Fig. 2 and 3).

### Heterogeneous effects.

Here and in the following studies we assessed mindsets—event-focused (growth mindset) and response-focused (stress-can-be-enhancing mindset)—via self-reports at baseline, and we conducted moderation analyses by these variables. Our objective was to reveal evidence about mechanisms ^48^, particularly concerning the complementarity of the two mindsets in shaping outcomes. We expected negative prior mindsets to predict worse stress responses in the control condition, and this was what we observed (see Extended Data Table 1). Further, we hypothesized that the synergistic mindsets intervention would differentially change stress response outcomes among participants who did not already endorse *both* positive mindsets, and who were therefore most at risk for negative outcomes. (We did not expect, and past mindset studies have not found ^26^, moderation for the immediate self-report outcomes such as those in Studies 1-2). Of note, using heterogeneity analyses such as these provides a more efficient use of the available statistical power than a 2 2 study crossing the two mindsets.

Because complex heterogeneity analyses carry a risk of yielding false-positive findings due to data snooping ^49,50^, we implemented an advanced and conservative statistical model called Bayesian Causal Forest (BCF) ^26,51^ to analyze all data. This method uses machine learning tools to model covariates (and their complex interactions), and also to model heterogeneous effects. It uses Bayesian Additive Regression Tree priors to make these models conservative. This avoids the problem of arbitrary covariate or moderator specifications leading to spurious results. Further, we summarized draws from the posterior distribution of effects ^52^, which avoids the risk for false positive results that comes from re-fitting models many times for each test of a simple effect, as might be done in a classical analysis ^53^.

The BCF heterogeneity analysis (see Fig. 3D) found that adolescents who held prior negative event and response-focused mindsets showed more threat-type physiological responding (e.g., higher TPR) during the stressful TSST epochs, a finding consistent with prior research ^54,55^ (also see Extended Data Table 1) Further, the treatment protected against a spike in TPR most strongly among these vulnerable young people with prior negative mindsets (Fig. 3D and 3E). Indeed, treated negative mindset individuals’ vascular resistance became indistinguishable from controls with positive prior mindsets (Fig. 3D). Thus, the intervention led to stress responses that typically follow from positive event- and response-focused mindsets and did so among the most vulnerable individuals (see also Extended Data Fig. 1). Analyses of heterogeneity for challenge-type stress responses (i.e. stroke volume) yielded the same pattern (Extended Data Fig. 2). Overall, the rather conservative heterogeneity analyses provided strong support for the theoretical model in Fig. 1.

## Effects on Internalizing Symptoms and Cortisol

Study 4 assessed the effects of the synergistic mindsets intervention on psychological and biological indicators of stress responses that have a longer time-course. Participants were adolescents attending a rigorous, urban public charter high school in a low-income neighborhood; 95% identified as Black/African-American or Hispanic/Latinx, and nearly all (99%) came from families facing economic disadvantages. This population was chosen because students facing the combination of socioeconomic disadvantages and high academic standards are likely to face chronic, daily stressors which have the potential to elicit threat-type stress responses ^5,56,57^, and could therefore stand to benefit from a stress optimization intervention. Also, it is important for samples in early-stage intervention research to be inclusive of the diversity of the population that, eventually, the intervention might be delivered to at scale.

**Figure 4:**
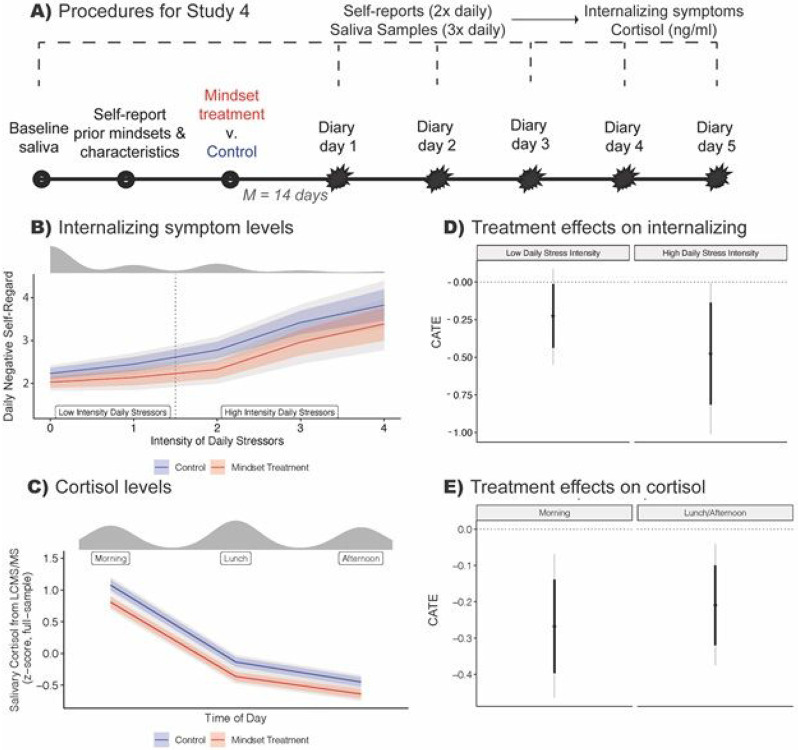
In Study 4 (N = 118, n <= 1213 observations), the synergistic mindsets intervention reduced internalizing symptoms overall and especially on intensely stressful days (B,D). The intervention also reduced daily salivary cortisol levels overall (C, E). Note: Starbursts represent stressor measurements. Univariate marginal distribution plots are over panels B and D. Thick / colored lines represent the 10th to 90th %iles; gray lines represent the 2.5th to 97.5th %iles. The vertical dashed line in (B) represents the cut-point for high vs. low daily stress intensity used to estimate subgroup CATEs in (D). In (D), the unstandardized CATE for high daily stress intensity was −.475 scale points [−.813, −.138]; for low daily stress intensity it was −.225 scale points [−.437, −.015].

The study procedures are depicted in Fig. 4A. Participants first completed a baseline survey assessment of prior negative mindsets, and then completed the intervention (or control) in a private room at school, with random assignment occurring at the individual level. Then, an average of 14 days later, students completed brief (5 min) daily stress surveys twice daily over the course of one week (4-5 consecutive school days), yielding up to 10 daily stress reports per individual. The daily surveys measured the intensity of evaluative stressors, and these were paired with self-reported internalizing symptoms. On the same days on which daily stress assessments were taken, students also provided up to three saliva samples (in the morning upon arrival at school, during lunch period, and after school ended) that were later assayed for cortisol levels using the LCMS/MS method ^58^.

When individuals are threatened by stressful events, cortisol levels exhibit large immediate increases, and these remain elevated after stress offset as the hormone lingers in the body for an hour or more ^42,47^. Cortisol levels assessed throughout the day can therefore be used to assess sustained HPA-axis engagement (i.e. threat-type stress responses) in naturalistic settings where daily stressors are not controlled and where there is not fine-grained stress onset or offset information. Affective states, by contrast, were assessed in reference to specific stressors that occurred prior that day. Thus, the two methods—daily symptom reports on intensely stressful days and overall cortisol levels—can provide complementary information about stress responses.

### Average effects: Internalizing symptoms.

The intervention reduced daily internalizing symptoms (i.e., feeling bad about oneself) overall by −.193 *SD* [−.331, −.049] compared to controls. As shown in Fig 4B, this effect was more than twice as large on highly stressful days, −.317 *SD* [−.542, −.092] relative to low stress days, −.150 *SD* [−.366, −.010]. This is consistent with our theoretical expectation of larger effects of the mindset intervention when people face the most demanding stressors, and it recalls the Study 3 finding of the largest effects during the most stressful speech epoch. As expected, daily stress intensity was positively associated with internalizing symptoms in the control condition, *r*(532) = .38, but this association was attenuated by 50% in the treatment condition, *r*(521) = .19 (Fig. 4B). Thus, the intervention achieved stress optimization, in that it promoted healthier, more resilient responses to intense, negative stressors. Like Studies 1-3, this study again demonstrates transfer of the intervention’s effects, this time to evaluative stressors occurring amidst the complexity of daily high school life.

### Heterogeneous effects: Internalizing symptoms.

The intervention’s effect on internalizing symptoms on high-stress days was 40% larger on average (−.38 *SD*) among individuals who endorsed both negative prior mindsets, relative to participants who endorsed positive prior mindsets (−.27 *SD*; see Extended Data Fig. 4). Overall, heterogeneity analyses again suggested that the intervention was counter-acting, in part, the two negative mindsets it was targeting.

### Average and heterogeneous effects: Cortisol.

There was an overall ATE on cortisol levels of −.227 *SD* [−.336, −.116], showing that the intervention attenuated HPA-axis activation. Self-reported daily stress intensity did not correlate with cortisol levels, *r*(1182) = .01, consistent with the interpretation that cortisol should be viewed as a measure of the functioning of the HPA system in response to persistent daily stressors, not only to specific daily stressors reported in the diaries. There was no meaningful heterogeneity (across time, stress intensity, or prior mindsets) in the cortisol effects.

## Effects on Overall Anxiety Symptoms

**Figure 5:**
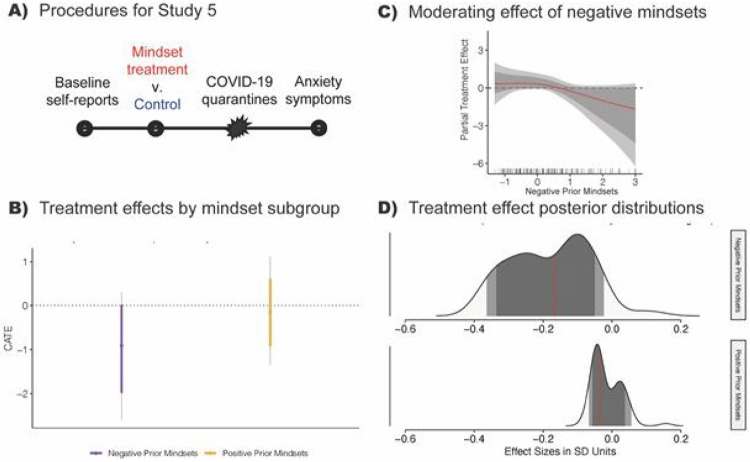
In Study 5 (N = 341) a synergistic mindsets intervention reduced general anxiety symptoms during the Spring 2020 COVID-19 quarantine among late adolescents with negative prior mindsets, and did not reduce anxiety among late adolescents with positive prior mindsets. (A) In January of 2020, participants answered prior negative mindset questions and completed the short mindset treatment or control exercise. Two and a half months later, during the first wave of stay-at-home orders during the COVID-19 pandemic in April of 2020, participants completed the Generalized Anxiety Disorder symptom assessment. (B) The treatment reduced anxiety symptoms by −.967 scale points [−2.086, .000] among those reporting negative prior mindsets and did not meaningfully reduce symptoms among those reporting positive prior mindsets, −.079 scale points [−.892, .738], (C) An additive summary of the posterior distribution of treatment effects shows greater reductions in anxiety in response to the treatment among those with negative prior mindsets. (D) Although there was a small posterior probability of a null treatment effect among prior negative mindsets participants, there was a higher probability of effects > .30 SD. The prior mindset subgroups used to display treatment effects in (B) and (D) were generated by implementing a hands-off Bayesian decision-making algorithm that maximized the differences among the mindset groups in terms of the outcome, without using information on the magnitudes of the treatment effects (see online supplemental material).

The effects on internalizing symptoms in Study 4 suggest the possibility for cumulative consequences for overall anxiety symptoms during times of negative stress (see Fig. 1). This possibility was tested with a new experiment. In Study 5, the environmental stressor was the forced exit from University housing, and subsequent social isolation and loneliness, during the early stages of the COVID-19 pandemic in the U.S in the Spring of 2020 (see study procedure in Fig. 5). The outcome of interest was scores on a standardized, widely-used screening tool for generalized anxiety symptoms ^59^. This is the same screening tool that has exhibited striking increases in generalized anxiety symptoms in representative sample surveys throughout the COVID-19 pandemic ^3^. Study 5 provided a strong test of the hypothesis that the young people in our experiment transferred the lessons from the intervention to cope with novel stressors, because we did not include, nor could have included, treatment messages relevant to the profound uncertainty and isolation that young people experienced during COVID-19 lockdowns. Because Studies 3 and 4 found stronger effects among those with negative prior mindsets—and because mindsets predicted greater anxiety in the control condition (Extended Data Table 1)— we expected the Bayesian algorithm to again find stronger effects for this group in Study 5.

Three months after the online intervention, we observed reductions in generalized anxiety symptoms, CATE = −.171 *SD*[−.371, .000], among adolescents who reported negative mindsets prior to random assignment (see Fig. 5B). Although the BCF model expected a small possibility of a null effect in this subgroup (see the top panel in Fig. 5D)—which is unsurprising because BCF uses a highly conservative prior distribution—the model concluded there was an even better chance of a treatment effect exceeding the threshold for a large real-world effect of .30 *SD* ^60^.

There was no discernible effect among adolescents with positive prior mindsets and who, as noted, were less likely to show anxiety symptoms in general, CATE = −.028 *SD* [−.173, .115] (also see Fig. 5D); that posterior distribution centered on zero and was largely non-overlapping with the negative prior mindsets group. Another way to interpret the moderation result is by plotting the additive relation between prior mindsets and the posterior distribution of treatment effects, holding other factors constant (Fig. 5C). Doing so showed that the intervention reduced anxiety symptoms more when they held stronger negative mindsets prior to random assignment.

## Discussion

Five experiments provided replicable evidence that a single-session, universal, self-administered, synergistic mindsets intervention lasting under an hour reduced internalizing symptoms and both cardiovascular and neuroendocrine indicators of threat-type stress responses. Because mindset interventions similar to the one tested here can be delivered cost-effectively in national or regional scale-up studies ^26,61^, the present research represents a critical theoretical step between basic insights about affect regulation and the discovery of actionable intervention methods that can produce real, lasting change at scale.

Remarkably, there was evidence that the short synergistic mindsets intervention reduced generalized anxiety symptoms during the COVID-19 lockdowns among participants whose mindsets presented a risk factor at baseline. Confidence in this subgroup conclusion was bolstered by (a) the use of a conservative, Bayesian, machine-learning modeling approach, and (b) the appearance of similar moderation for threat-type stress physiology and internalizing symptoms in other studies. In addition to its practical relevance, this subgroup finding is informative for theory. It suggests that the intervention works by interrupting the negative recursive process^62,63^ of appraisals stemming from negative mindsets that, left unchecked, could have accumulated negative psychological consequences (also see the top-right in Fig. 1B).

We emphasize that the present intervention applies only to plausibly beneficial stressors, such as formal schooling, but not all stressful experiences can or should be appraised positively. Trauma or abuse, for instance, are unambiguously negative and usually uncontrollable. Even so, people who have experienced chronic environmental stressors may be still helped by the present mindset intervention if they could apply it to their normative stressors (see Studies 4 and 5).

Careful attention to heterogeneity of effects will be required to make the synergistic mindsets intervention ready for wider-scale implementation.^64^ First, the materials would need to be extensively adapted for other stressful contexts that are different from the academic contexts studied here ^65^. Second, mindset interventions in general depend on the supports, or *affordances*, in a context to sustain the self-reinforcing cycles that propagate their effects overtime (Fig. 1) ^26,66,67^. Currently, we do not know which affordances are most critical, or how to make them more abundant; such knowledge will be a high priority for the next phase of research.

Finally, the present evidence is aligned with an emerging perspective on adolescence ^1,15,68-70^. This perspective emphasizes adolescents’ potential to agentically shape their own positive development, rather than focusing on adolescence as an inherently risky stage. It also encourages policymakers to build on the strengths of adolescents—for instance, their abilities to optimize their stress responses—rather than infantilizing or pathologizing them ^11,15,68-70^. Consistent with this view, here we showed that negative trajectories of stress and internalizing symptoms were not inevitable, and could be addressed through empowering messages. This suggests that future research could examine how cultural frames and narratives about adolescents, and the stresses they encounter, might be optimized. Doing so may help to promote thriving at a societal scale.

## Methods

### Ethics approval.

Approvals for these studies were obtained from the Institutional Review Boards at the University of Rochester or the University of Texas at Austin.

### Pre-registration and efforts to curb researcher degrees of freedom.

Studies 1 and 2 were preregistered (osf.io/tgysd; ^55^). Studies 3 and 4 limited degrees of freedom by following published and previously preregistered standard operating procedures for TSST and daily diary studies conducted by the labs carrying out this research ^45^, (the focus on TPR, SV, and PEP in Study 3 and the focus on the stressor intensity ‘ treatment interaction in Study 4). Study 5, which focused on anxiety symptoms during the COVID-19 lockdown, was not preregistered because the pandemic was unanticipated. Researcher degrees of freedom were limited by following the same analysis steps (covariates, moderators, and BCF modeling) as Studies 1-4 whenever possible.

### Intervention overview.

The intervention consisted of a single self-administered online session lasting approximately 30 minutes. Random assignment to the intervention or control condition occurred in real-time via the web-based software, as participants were completing the online intervention materials. Participants were blinded to the presence of different conditions, and teachers or others interacting with participants were blind to the intervention content and to condition assignment. Thus, the intervention experiments used a double-blind design throughout.

### Synergistic mindsets intervention.

The intervention used methods for mindset interventions that are well-established in the literature and have been used successfully in national scale-up studies ^26^. The intervention first aimed to convey the message that stressful events are controllable and potentially helpful. It did so by reducing negative fixed mindset beliefs, or the belief that intellectual ability is fixed and cannot change, which can lead to the appraisal that negative events are uncontrollable and harmful. In particular, the fixed mindset leads to a pattern of appraisals about effort (that having to try hard or ask for help means you lack ability), about causes of failures (the attribution that failure stems from low ability), and about the desired goal in a setting (the goal of not looking stupid in front of others) ^39,71^. The intervention overcame these negative patterns of appraisals by conveying the growth mindset. The growth mindset promotes the appraisal that difficulties can be controlled and helpful. It argues that most people who became good at something important had to face and overcome struggles, and therefore, your own struggles should not be viewed as signs of deficient abilities but instead should be viewed as part of your path toward important skill development. To justify the controllable/helpful stressor appraisal, the intervention drew on neuroscientific information about the brain’s potential to develop more efficient (i.e., “stronger”) connections when it faces and overcomes challenges, using the analogy of muscles growing stronger when they are subjected to rigorous exercise ^29^.

Second, the intervention aimed to reduce the stress-is-debilitating mindset ^22^, which is the belief that stress is inherently negative and compromises performance, health, and wellbeing; this mindset leads to the appraisal that a given stressor is uncontrollable and harmful. Counter to the stress-is-debilitating mindset, the intervention developed here introduced the stress-can-be-enhancing mindset^22^, which is the belief that stress can have beneficial effects on performance, health, and wellbeing; this more adaptive belief system leads to the appraisal that stressors can be potentially helpful and controlled. The intervention explained that when people undergo challenges, they inevitably begin to experience stress, which can manifest in a racing heart, sweaty palms, or possibly feelings of anxiety or worry. The intervention leads people to perceive those signals as information that the body is preparing to overcome the challenge, for instance by providing more oxygenated blood to the brain and the muscles ^33^. Thus, the stress response is framed as helpful for goal pursuit, not necessarily harmful. The intervention also argued that feelings of anxiety can be a sign that you have chosen a meaningful and ambitious set of goals to work on, and therefore can indicate a positive trajectory, not a negative one.

Importantly, these two mindsets were conveyed *synergistically*, not independently, so that they built on one another. Participants were encouraged to view struggles as potentially positive and worth engaging with, and then they were invited to view inevitable stress coming from this engagement as a part of the body’s natural way to help them overcome the stressor.

These mindset messages were couched within a summary of scientific research on human performance and stress. Participants were not simply informed of these facts, but they were instead invited to engage with them, make them their own, and plan how they could use them in the present and future.

Participants heard stories from prior participants (older students in this case) who used these ideas to have success in important performance situations, and they also completed open-ended and expressive writing exercises. For instance, participants wrote about a time when they were worried about an upcoming stressor, and then later on they wrote advice for how someone else who might be undergoing a similar experience could use the two mindsets they learned about—which has been called a “saying-is-believing” writing exercise ^72^.

### Control group content.

The control group intervention was also an online, self-administered activity lasting ~30-minutes. It was designed to be relatively indistinguishable from the intervention group by using similar visual layout, fonts, colors, and images. The content was predominately from the control condition from a prior national growth mindset experiment ^26^, which included basic information about the brain and human memory. It also involved open-ended writing activities and stories from older students. However, the control condition did not make any claims about the malleability of intelligence. To this standard content we added basic information about the body’s stress response system (e.g., the sympathetic and parasympathetic nervous system and the HPA-axis) to control for the possibility that simply reflecting on stress and stress responses could account for the results. The latter content did not include any evaluations of whether stress responses are good or bad, or controllable or uncontrollable.

### Negative prior mindsets.

At baseline, participants in all experiments except Study 2 completed standard, three-item measures of negative event-focused mindsets (fixed mindset of intelligence, i.e., “Your intelligence is something about you that you can't change very much.”) ^26^ and response-focused mindsets (the stress-is-debilitating mindset ^14^, i.e. “The overall effect of stress on my life is negative.”) (for both, 1 = *Strongly disagree*, 6 = *Strongly agree*). In the primary Bayesian analyses, the two measures and their product were entered into the covariate and moderator function, and the machine-learning algorithm decided how best to use the mindset measures to optimize prediction or moderation. In the preliminary correlational analyses (Extended Data Table 1), we analyzed the multiplicative term of the two, for simplicity.

#### Analysis strategy

For all experimental analyses, we used intention-to-treat analyses, which means that data were analyzed for all individuals who were randomized to condition and who provided outcome data, regardless of their fidelity to the intervention protocol. This analysis is more conservative but also better reflects real-world effect sizes.

The present research advanced a fully-Bayesian regression approach called targeted, smooth Bayesian Causal Forest (tsBCF or BCF) ^73^ to calculate treatment effects and understand moderators of the treatment effects. A previous version of the BCF algorithm has won several open competitions for yielding honest and informative answers to questions about the complex, but systematic, ways in which a treatment’s effects are, or are not, heterogeneous, and it is designed to be quite conservative ^51^. We used the existing BCF method for Studies 1, 2, 4 and 5. The model is specified in Eq. 1:

(1) $$y_{ij}=\alpha_{i}+\beta (x_{ij})+\tau (w_{ij})\cdot z_{i}+\epsilon_{ij}$$

In Study 3 we updated the BCF method to apply to multilevel, time series data. See Eq. 2:

(2) $$y_{ij}=\alpha_{j}+\beta (x_{j},t_{ij})+\tau (w_{ij},t)z_{j}+\epsilon_{ij}$$

In Eq. 1 and 2, *y_ij_* is the outcome for adolescent *i* at time *j*, *α_j_* is the random intercept for each individual, *x_j_* is the vector of covariates which predict the outcome and could control for chance imbalances in random assignment, *w_ij_* is the vector of potential treatment effect moderators, *t* is time (the *t* term is omitted in all studies except Study 3), *z_j_* is the dichotomous treatment effect indicator for each individual, and *ε_ij_* is the error term. What makes BCF unique, and well-suited for this application, is that both *β*(.) and *τ*(.), are non-linear functions that take a “sum-of-trees” representation, and which are estimated using machine learning tools. This frees researchers from making arbitrary decisions about which covariates to Include, what their functional form should be, and how or whether covariates should interact.

Notably, BCF uses conservative prior distributions, especially for the moderator function, to shrink toward homogeneity and to simpler functions, avoiding over-fitting. The data are used once—to move from the prior to the posterior distribution—and all analyses then summarize draws from the posterior. This approach contrasts with the classical method, which involves re-fitting the model many times to estimate simple effects or to conduct robustness analyses with different specifications. The BCF approach, therefore, reduces researcher degrees of freedom, mitigating the risk of false discoveries and other spurious findings. In this research we focused on estimation of treatment effects (i.e. how large the effect is) and not null-hypothesis testing (i.e. whether it is “significant” or not) because of well-known problems with the all-or-nothing thinking inherent in the null hypothesis significance test ^74^. Following convention^75^ we reported the average treatment effects (ATE) and the conditional treatment effects (CATEs) with the associated 10^th^ and 90^th^ %iles from the posterior distributions (see Figures for the 2.5 and 97.5 %iles).

### Effect size calculations.

Unless otherwise noted, effects are standardized by the raw *SD* in the control condition.

### Manipulation checks (all studies).

The intervention reduced negative mindset beliefs (four items, including “*Stress stops me from learning and growing*” and “*The effects of stress are bad and I should avoid them*”, 1 = *Strongly disagree*, 6 = *Strongly agree*). Analyses revealed lower levels of negative mindsets in the intervention condition at post-test compared to the control condition, signifying a successful manipulation check: Study 1) −.293 *SD* [−.426, −.161]; Study 2) −.437 *SD* [−.567, −.310]; Study 3) −.504 *SD* [−.724, −.504]; Study 4) −.255 *SD* [−.549, .030]; Study 5) −.556 *SD* [−.713, −.399], The two field experiments with high schoolers (Studies 1 and 4) had smaller manipulation check effects that were more imprecise than the others (Studies 2, 3, and 5). This was expected because the former studies were conducted in naturalistic school settings that tend to produce noisier data.

#### Study 1

##### Sample size determination.

Sample size was planned to have sufficient power to detect a treatment effect in a field experiment of .10 *SD* or greater, with .10 *SD* being the minimum effect size that we would interpret as meaningful for a study focused on immediate post-test self-reports. We worked with our data collection partner, the Character Lab Research Network (CLRN) ^76^, to recruit as close to 3,000 participants as possible in a single semester. The final sample size was determined by the logistical constraints of data collection during the COVID-19 pandemic.

##### Participants.

Participants were a heterogeneous national sample of adolescents who were evenly distributed across grades 8 to 12 in U.S. public schools (13 y/o: 16%; 14: 20%; 15: 20%; 16: 21%; 17:18%; 18: 5%). Forty-nine percent identified as male, 49% as female, and 2% as gender non-binary. Participants were also racially and ethnically diverse (participants could indicate multiple racial/ethnic identities so numbers exceed 100%): Black: 20%; Latinx: 39%; White: 68%; Asian: 7%. Participants were also socioeconomically diverse: 40% received free or reduced price lunch, an indicator of low family income. Therefore, Study 1 provided a test of the hypothesis that the intervention could be widely disseminated and effectively change beliefs and appraisals in a national sample of adolescents that reflected the diversity of students in U.S. public schools. Even so, the sample was not strictly nationally representative because random sampling was not used to recruit the CLRN sample.

##### Procedure.

Participants were recruited by CLRN^76^, which administers roughly 45-minute online survey experiments three times per year to a large panel of adolescents attending 6^th^ to 12^th^ grade. Researchers program their studies using the Qualtrics platform and students self-administer the materials at an appointed time. Data collection continued during the modified instructional settings of Fall 2020. We note that all measures had to be short so as to keep respondent burden low and fit within the required time limit for CLRN studies. Thus, the tradeoff in Study 1, when achieving scale and reaching a large adolescent population during the COVID-19 pandemic, was estimating potentially weaker effect sizes due to statistical noise.

##### Measures.

At the beginning of the survey, participants indicated their most stressful class (e.g., math, science, English / Language arts). Then, after the intervention (or control) experience they were asked to imagine that “later today or tomorrow your teacher [in your most stressful class] asked you to do a very hard and stressful assignment. Imagine this is the kind of assignment that will take a lot of time to finish but you only have two days to turn it in. Also pretend that you will soon have to present your work in front of the other students in your class.” Participants then reported their event-focused appraisals on three items (e.g., “*How likely would you be to think that the very hard assignment is a negative threat to you?*”, 5 = *Not at all likely to think this*, 1 = *Extremely likely to think this*). Next participants reported their response-focused appraisals (“*Do you think your body's stress responses (your heart, your sweat, your brain) would help you do well on the assignment, hurt your performance on the assignment, or not have any effect on your performance either way?*” 5 = *Definitely hurt my performance*, 1 = *Definitely help my performance*).

The end of the study also included an additional behavioral intention measure: a choice between an “easy review” extra credit assignment and a “hard challenge” assignment ^61,65^. The intervention increased the rate of choosing the challenging assignment by .11 *SD* [.028, .200], We expected the treatment to increase engagement with stressors because it leads to the appraisal that they are opportunities for learning and growth.

#### Study 2

##### Sample size determination.

All students in an introductory social science course in Fall 2019 were asked to complete the intervention or control materials. Sample size was set by the response rate.

##### Participants.

Participants were predominately first-year college students attended a selective public university in the United States that draws from a wide range of socioeconomic status groups: 17 years-old: 3%; 18: 49%; 19: 29%; 20: 11%: 21 or older: 8%. Sixty-four percent identified as female and the rest as male; 39% had mothers who did not have a four-year college degree or higher (an indicator of lower socioeconomic status), and 59% identified as lower class, lower middle class, or middle class (vs. upper middle or upper class).

##### Procedure.

This experiment was conducted in a social science course in which students completed timed, challenging quizzes at the beginning of each class meeting, twice per week. In the second week of the semester, soon before the first graded quiz, students were invited to complete the intervention (or control) materials on their own time using their own computer in return for course credit, and 83% of invited students did so. The effects of the intervention were assessed via students’ appraisals of the first graded quiz of the semester one to three days later. The appraisal items were necessarily short because they were embedded at the end of the assignment and students completed them during class before the lecture. The appraisal items were then administered a second time after another quiz which occurred 3-4 weeks post-intervention.

##### Measures.

Participants rated their agreement or disagreement with the statements “*I felt like my body’s stress responses hurt my performance on today’s benchmark*” (1 = *Strongly disagree*, 5 = *Strongly agree*) and “*I felt like my body’s stress responses helped my performance on today’s benchmark*” (5 = *Strongly disagree*, 1 = *Strongly agree*). The two ratings were averaged to provide an appraisal index, with higher values corresponding to more negative appraisals ^77^.

#### Study 3

##### Sample size determination.

An *a priori* power analysis was used to determine sample size. Previous stress research that assessed cardiovascular responses in laboratory-based stress induction paradigms produced medium to large effect sizes (e.g., range: *d* = .59 to *d* = 1.44 in Yeager et al., 2016, Jamieson et al., 2012, Oveis et al., 2020). Based on a standard medium effect size, at the low end of this range (*d* = 50), G*Power indicated that 64 participants per condition (i.e., 128 total participants) would be necessary to achieve a target power level of .80 to test for basic effects of the treatment using frequentist methods. In anticipation of potential data loss, we determined *a priori* that we would oversample by 20%. Data collection was terminated the week after more than 150 participants were enrolled in the study.

##### Participants.

Participants were prescreened and excluded for physician-diagnosed hypertension, a cardiac pacemaker, BMI > 30, and medications with cardiac side effects (e.g., Blascovich et al., 2011). A total of 166 students were recruited from a university social science subject pool (120 females, 46 males; 76 White/Caucasian, 12 Black/African-American, 17 Latinx, 65 Asian/Asian-American, 2 Pacific Islander, 4 Mixed Ethnicity, 7 Other; *Mage* = 19.81, *SD* = 1.16, *range* = 18–26). After data collection, two participants were excluded due to experimenter errors. Additionally, impedance cardiography data for four participants could not be analyzed due to technical issues (prevalence of noise and artefacts in the signals). Decisions about inclusion of participants were made blind to condition assignment and to levels of the outcome. Participants were compensated $20 or 2-hrs of course credit for their participation.

##### Procedure.

After intake questions, application of sensors, and acclimation to the lab environment, participants rested for a 5-min baseline cardiovascular recording which occurred approximately 25-min after arrival at the laboratory. They were then randomly assigned to an intervention condition by the computer software in real time and completed either intervention or control materials, which took approximately 20 minutes in this sample. Participants then completed the Trier Social Stress Test (TSST) ^44^. The TSST asks participants to give an impromptu speech about their personal strengths and weaknesses in front of two evaluators. Evaluators are presented as members of the research team who are experts in nonverbal communication and will be monitoring and assessing the participant’s speech quality, ability to clearly communicate ideas, and nonverbal signaling. Throughout the speech (and math) epochs of the TSST, evaluators provide negative nonverbal feedback (e.g., furrowing brow, sighing, crossing arms, etc.) and no positive feedback, either nonverbal or verbal ^44^. At the conclusion of speeches, and without prior warning, participants are asked to do mental math (counting backwards from 996 in increments of 7) as quickly as possible in front of the same unsupportive evaluators. Incorrect answers were identified by evaluators, and participants were instructed to begin back at the start. This stress induction procedure is widely used to induce the experience of negative, threat type stress responses ^45,47^. After completion of the TSST task, participants rested quietly for a 3-min recovery recording, and prior to leaving the lab all participants were debriefed and comforted.

##### Physiological Measures.

The following measures were collected during baseline and throughout the Trier task: electrocardiography (ECG), impedance cardiography (ICG), and blood pressure (BP). ECG and ICG signals were sampled at 1000 Hz, and integrated with a Biopac MP150 system. ECG sensors were affixed in a Lead II configuration. Biopac NIC00100C cardiac impedance hardware with band sensors (mylar tapes wrapped around participants’ necks and torsos) were used to measure impedance magnitude (Zo) and its derivative (dZ/dt). BP readings were obtained using Colin7000 systems. Cuffs were placed on participants' non-dominant arm to measure pressure from the brachial artery. BP recordings were taken at 2-min intervals during baseline, throughout the stress task, and recovery. BP recordings were initiated from a separate control room. ECG and ICG signals were scored offline by trained personnel. First, one-minute ensemble averages were analyzed using Mindware software IMPv3.0.21. Stroke volume (SV) was calculated using the Kubicek method ^78^. B- and X-points in the dZ/dt wave, as well as Q- and R-points in the ECG wave, were automatically detected using the maximum slope change method. Then, trained coders blind to condition examined all placements and corrected erroneous placements when necessary.

Analyses targeted three physiological measures: pre-ejection period (PEP), stroke volume (SV), and total peripheral resistance (TPR). This suite is commonly used to threat-type stress responses (for a review see ^79^). TPR is the clearest indicator of threat-type responses and was therefore the focal outcome measure in this research. TPR assesses vascular resistance, and when threatened, resistance increases from baseline ^43^. TPR was calculated using the following validated formula: (MAP / CO) * 80 ^80^. PEP is a measure of sympathetic arousal and indexes the contractile force of the heart. Shorter PEP intervals indicate greater contractile force and sympathetic activation. Both challenge and threat type stress responses are accompanied by decreases in PEP from rest. SV is the amount of blood ejected from the heart on each beat (on average per minute). Increases in SV index greater beat-to-beat cardiac efficiency and more blood being pumped through the cardiovascular system, and are often observed in challenge states ^45^. Decreases in SV, on the other hand, are more frequently observed in threat states (even though threat can also elicit little or no change in SV ^81^). Cardiac output (CO), which is SV multiplied by heart rate (HR), is frequently used to assess threat and challenge type stress responses as well. As in a past paper^45^ we focused on SV rather than CO because the effects of the treatment on PEP (and thus HR) during the recovery period could distort effects on CO.

#### Study 4

##### Sample size determination.

The number of students recruited each week was constrained by the research team’s capacity to support twice-daily diary surveys and thrice-daily saliva samples in a school environment. The ultimate sample size was determined by the total number of students who could be recruited from the school in the fall semester of 2019 given these constraints.

##### Participants.

Participants were adolescents from economically-disadvantaged families who were nearly all (95%) from black or indigenous racial/ethnic groups. Students attended a high-quality urban charter school which showed a high graduation rate (98%) relative to the urban city school district (68%). Therefore, this was a population that was expected to face social, economic, and academic stressors, and who could therefore make use of a stress optimization intervention.

##### Procedure.

Participants were assigned to one of three data collection cohorts based on their academic schedules and available research staff. Cohorts 1, 2, and 3 completed daily diary measures across three consecutive weeks during the Fall term. The intervention was administered on a Thursday, and then students began their weekly daily diary data collection 1-3 weeks later (*M* = 14 days). Intervention materials (see Experiment 1) were completed on a tablet computer with headphones in a quiet room at the school. Randomization to conditions occurred at this time. All instructors and research staff were blind to condition assignment and specific hypotheses. Prior to intervention/control materials participants completed baseline measures of mindsets (stress mindsets and growth mindsets) along with demographic information.

The week of daily diary data collection began on a Monday and students were surveyed twice each day for five consecutive days through Friday. Students provided their first report at lunch and the second at the conclusion of the school day but before leaving the school’s campus. Saliva samples were collected three times per day by adding the morning, before the first class period of the day. Thus, we targeted 10 total reports for each student and 15 total saliva samples. In addition to occasional non-response, there were two exceptions to these targeted numbers. One cohort had four days of data collection due to a school-wide event on a Friday, and the first cohort had up to three preliminary days of self-report (not saliva) data collection while the research team was refining procedures. Rather than exclude these additional self-report records, they were included, although the results were the same when excluding them.

The daily diary measures were designed to be brief (~5 min) and were completed on paper. When reporting on daily stressful events, students first indicated the categories of stressors they experienced that day (e.g., friends/social, academics, romantic relationships, daily hassles, etc.), then how intense the stressors, combined, were overall (“*How negative would you say these experiences were?*” 1 = *Not negative at all*, 5 = *Extremely negative*). Following published standard operating procedures for the diary studies in this lab^45^, days on which no social-evaluative stressors were listed were coded as a “1” for stressor intensity (the lowest value), to avoid dropping data from analysis.

Students were compensated $10 for completing intervention materials, and $5 for each daily diary entry. Thus, the maximum compensation per participant was $60. After the conclusion of data collection, students and instructors were debriefed, and students randomly assigned to the control condition were provided with the mindset intervention.

##### Internalizing symptoms.

On each daily survey, students reported internalizing symptoms, operationalized as overall positive or negative feelings about themselves (“*Overall, how good or bad did you feel about yourself today?*” 1 = *Extremely good, 7 = Extremely Bad*).

##### Cortisol.

Acute cortisol responses follow a specific time course (i.e. peak levels occur ~30 minutes after stress onset). However, the diary survey stressors were not calibrated to identify the timing of specific events, so the two sources of information could not be tightly yoked. Indeed, as noted in the main text, there was no association between intensity of stressors reported and cortisol in the control condition. This is in contrast to the relation between internalizing symptoms and stressor intensity in the control condition. Additionally, cortisol levels have a diurnal cycle (i.e., peak levels at wakening, rapid declines within the first waking hours, and nadir at the end of the day). Waking levels and diurnal slopes can map onto wellbeing, stress coping, and health ^82^. Because all sampling was conducted during the school day, waking levels and diurnal cortisol slopes could not be accurately and precisely measured. The lack of time-course specificity and diurnal cycle data meant that our reported effect sizes for global cortisol levels are likely conservative.

#### Study 5.

##### Sample size determination.

We recruited all students possible from an entire social science class in the spring of 2020, which, we would later learn, was a unique cohort for examining stress during the COVID-19 lockdowns.

##### Participants, procedure, and measures.

Data were collected during the Spring semester of 2020. Participants were from the same university as Study 2 and the same intervention procedures were followed. (Due to a difference in data collection procedures relative to Study 2, quiz appraisal data could not collected in Study 5). The intervention was delivered at the end of January 2020. In March of 2020, students were sent home due to COVID-19 quarantines. In mid-April of 2020, students completed the General Anxiety Disorder-7 (GAD-7)^59^ as a part of a class activity focused on psychopathology. The GAD-7 asks “*How often have you been bothered by the following over the past 2 weeks?*” and offers several symptoms, including “*Feeling nervous, anxious, or on edge*” “*Not being able to stop or control worrying*,” and “*Feeling afraid as if something awful might happen.*” Each symptom is rated on a scale from 0 = *Not at all* to 3 = *Nearly every day*. The seven items were averaged, producing an overall score with higher values corresponding to higher levels of general anxiety symptoms.

## Supplementary Material

Supplement 1

Supplement 2

Supplement 3

## Figures and Tables

**Table 1.** Overview of studies

Study (Sample Size)	Population	Stressor	Measure of Threat-Type Stress Response
1 (*N* = 2,717)	13-18 y/o U.S. public school students during the COVID-19 pandemic.	Anticipated timed assignment	Event- and response-focused appraisals
2 (*N* = 755)	Diverse undergraduate students attending a public university	Experienced timed assignment	Cognitive appraisals at 1-3 days and 3 weeks post-test
3 (*N* = 160)	Undergraduate students at a private university	Trier Social Stress Test	Peripheral blood flow
4 (*N* = 118, *n*=1213 observations)	14-16 y/o adolescents from racial/ethnic minority groups, facing economic disadvantages	Daily stressors in high school	Daily internalizing symptoms and HPA-axis activation (cortisol)
5 (*N* = 341)	Same as study 2 but during the onset of the COVID-19 pandemic in Spring 2020	COVID-19 quarantines	Generalized internalizing symptoms

Note: Across the five experiments, the synergistic mindsets intervention reduced maladaptive beliefs compared to the control condition by .25 *SD* or greater, which means each experiment passed the manipulation check (see the methods for greater detail).

## Data Availability

Student-level data are protected by federal laws and data sharing agreements with the partnering institutions; de-identified data can be accessed by researchers who agree to terms of data use, including required training and approvals from the University of Texas Institutional Review Board and analysis on a secure server at no cost to researchers. To request access to data, researchers should contact mindset@prc.utexas.edu. Researchers wishing to access full intervention materials can find them in the supplemental materials.
