# Supplementary material for: A Synergistic Mindsets Intervention Protects Adolescents from Social Stress
Source: Res Sq. 2021 May 28:rs.3.rs-551170. Preprint. [Version 1] doi: 10.21203/rs.3.rs-551170/v1 (PMC8168396; doi:10.21203/rs.3.rs-551170/v1)
Supplement: Supplement 1 [file 76d10246a0b3d771668cf3a8.docx]

## Details of the Bayesian Causal Forest (BCF) Analysis

As noted in the paper, BCF has been found, in multiple open competitions and simulation studies, to detect true sources of treatment effect heterogeneity while not lending much credence to noise ^1–3^. BCF builds on the popular Bayesian Additive Regression Trees (BART)^4^ approach. Both Bayesian regression tree models and BCF in particular have been top performers in empirical evaluations of methods for causal inference ^2,3,5,6^. Here we provide more details about how the BCF model was specified and estimated.

In the BCF analysis for studies 1, 2, 4, and 5, the model is specified as

$$\begin{matrix} y_{ij}=\alpha_{i}+\beta\left( x_{ij} \right)+\tau\left( w_{ij} \right)\cdot z_{i}+\epsilon_{ij}, \end{matrix}$$

where $y_{ij}$ is outcome *j* for student *i* and $z_{i}$ is a treatment assignment indicator. Here $x$ is a vector of covariates and $w$ is subset of these which are potential treatment effect moderators. These covariates may be measured at the individual level or the observation level. We also allow for individual-level intercept random effects, $\alpha_{i}$, to account for varying levels and clustering due to repeated observations in Study 4. The observation-level error term $\epsilon_{ij}$ is assumed to be normally distributed with variance $\sigma^{2}$.

Here $\beta$ and $\tau$ are nonparametric functions which allow for nonlinearities and interactions between covariates in affecting the expected outcome and treatment effects. This model specification is similar to traditional multilevel linear models of heterogeneous treatment effects, but relaxes the strict assumption of linearity and additivity between the covariates and the expected value of the outcome and conditional average treatment effects.

For study 3, the BART priors on $\beta$ and $\tau$ are replaced by *targeted smooth* BART priors, introduced in ^7,8^, with time as the smooth variable. These priors are described below. The time series model for Study 3 is specified as

$$y_{it}=\alpha_{i}+ \beta\left( x_{j}, \right)+\tau\left( w_{ij}, \right)z_{i}+\epsilon_{it}$$

**Prior specification**

To complete our Bayesian model, we must specify prior distributions for the unknown parameters in the equations above. These include the nonparametric functions $\beta(\cdot)$ and $\tau(\cdot)$, the random effects $\alpha_{i}$, and the error variance $\sigma^{2}$.

The priors for the functions $\beta(\cdot)$ and $\tau(\cdot)$ in studies 1, 2, 4, and 5 are taken from the Bayesian causal forests model (BCF; Hahn et al., 2020). Under this model, both functions have a sum-of-trees representation, as first defined for Bayesian methods in Chipman, George, and McCulloch (2010). Each tree consists of a set of internal decision nodes which partitions the covariate space, and a set a of terminal nodes, or leaves, corresponding to each element of the partition. The prior for each of $\beta(\cdot)$ and $\tau(\cdot)$ is comprised of three parts: the number of trees, two parameters controlling the depth of each tree, and a prior on the leaf parameters. Use of this sum-of-trees term allows for detection of nonlinearity and interactions between covariates. The prior for the functions $\beta(\cdot)$ and $\tau(\cdot)$ in study 4 is similar, except it replaces the constant predictions of a BART prior with generic functions of time, assigned Gaussian process prior distributions calibrated as in ^7,8^.

A key feature of the BCF model is that the prior for $\tau(\cdot)$, which captures heterogeneity in the intervention effect, is regularized more heavily compared to the control function $\beta(\cdot)$ in order to shrink toward homogeneous effects, i.e. that the intervention effect is constant across all values of the moderators. The prior for $\tau(\cdot)$ uses fewer trees, with each tree being regularized to be shallower (that is, contain fewer partitions). Details are on prior specification are given in ^1,4,7,8^.

The random effect $\alpha_{j}$ is given a Gaussian prior with the standard deviation having a prior of a half $t$-distribution with 3 degrees of freedom, as recommended by Gelman^9^. Finally, the error variance is given an inverse chi-squared prior with 3 degrees of freedom and scale parameter informed by the data.

**Posterior Inference and Summarization**

To conduct inference we sample from the posterior distribution of the model parameters using a Markov chain Monte Carlo (MCMC) sampling scheme. MCMC sampling for Bayesian sum-of-tree models is described in ^1,4,8,10^. Draws from the posterior distribution can then be aggregated to derive posterior distributions for average treatment effects, subgroup average treatment effects, other conditional average treatment effects, or other aggregates or summaries of the model parameters.

We defined subgroups of participants with negative and positive prior mindsets based on their expected outcomes in the absence of our treatment, which is captured by the $\beta(x_{ij})$ term in our model. We treat choosing subgroups as a Bayesian decision problem. We defined a class of possible subgroups based on threshold values of the prior growth mindset and prior stress-can-be-enhancing scales (Fig. S1). We chose as a utility function the difference in average outcomes under control between the two groups, so that positive and negative prior mindset groups correspond to those with favorable and unfavorable outcomes in the absence of the intervention. Solving this decision problem entails an exhaustive search for the thresholds that best separate the subgroup average outcomes under control conditions, subject to a minimum sample size of 10.

Finally, to understand the *partial* or *adjusted* effects of some effect moderators—their influence on the estimated treatment effect function when the other moderators are held constant—we use additive spline summaries (or generalized additive models^11^), as described in ^12^. The BCF estimate of $\tau$ in studies 3-5 was approximately additive (and we found no heterogeneity in Studies 1-2, as expected). Therefore, to give an interpretable estimate of this conditional intervention effects in Studies 3-5, we created an additive summary of the fitted $\tau(\cdot)$ function using splines, and looked at the partial effect of prior negative mindsets, conditional on alternative potential moderators**.** This additive summary closely tracks the fitted $\tau$ function itself, so this additive summary is a faithful recapitulation.

Positive Prior Mindsets

Prior growth mindsets

aa

b

Negative Prior Mindsets

Prior stress-can-be-enhancing

c

d

Fig. **S1**. *Graphical representation of positive and negative prior mindset subgroups.* The positive and negative prior mindsets subgroups are defined by four cutoffs *a,b,c,* and *d*. An adolescent is in the positive prior mindsets group if their scores on the prior growth mindset scale and prior stress-can-be-enhancing scale are above *a* and *d*, respectively, with the negative prior mindsets group defined similarly. The subgroups are defined by searching over the cutoffs to find those which maximize the difference in average outcomes under the control condition between the two subgroups – as estimated by the model – subject to minimum sample sizes in each groups. Note that this process occurs automatically with an unsupervised algorithm, in that researchers do not define the cutpoints *a, b, c,* or *d* by hand. This avoids researcher degrees of freedom concerning the cutpoints chosen.

**References**

1. Hahn, P. R., Murray, J. S. & Carvalho, C. M. Bayesian regression tree models for causal inference: regularization, confounding, and heterogeneous effects. *Bayesian Analysis* (2020) doi:10.1214/19-BA1195.

2. McConnell, K. J. & Lindner, S. Estimating treatment effects with machine learning. *Health Services Research* **54**, 1273–1282 (2019).

3. Wendling, T. *et al.* Comparing methods for estimation of heterogeneous treatment effects using observational data from health care databases. *Statistics in medicine* **37**, 3309–3324 (2018).

4. Chipman, H. A., George, E. I. & McCulloch, R. E. BART: Bayesian additive regression trees. *The Annals of Applied Statistics* **4**, 266–298 (2010).

5. Dorie, V., Hill, J., Shalit, U., Scott, M. & Cervone, D. Automated versus do-it-yourself methods for causal inference: Lessons learned from a data analysis competition. *Statist. Sci.* **34**, 43–68 (2019).

6. Hahn, P. R., Dorie, V. & Murray, J. S. Atlantic Causal Inference Conference (ACIC) Data Analysis Challenge 2017. *arXiv preprint arXiv:1905.09515* (2019).

7. Starling, J. E. *et al.* Targeted Smooth Bayesian Causal Forests: An analysis of heterogeneous treatment effects for simultaneous versus interval medical abortion regimens over gestation. *arXiv:1905.09405 [stat]* (2020).

8. Starling, J. E., Murray, J. S., Carvalho, C. M., Bukowski, R. K. & Scott, J. G. Bart with targeted smoothing: An analysis of patient-specific stillbirth risk. *Annals of Applied Statistics* **14**, 28–50 (2020).

9. Gelman, A. Prior distributions for variance parameters in hierarchical models (comment on article by Browne and Draper). *Bayesian Anal.* **1**, 515–534 (2006).

10. Hill, J., Linero, A. & Murray, J. Bayesian Additive Regression Trees: A Review and Look Forward. *Annual Review of Statistics and Its Application* **7**, 251–278 (2020).

11. Hastie, T. & Tibshirani, R. *Generalized additive models*. (Chapman and Hall, 1990).

12. Woody, S., Carvalho, C. M. & Murray, J. S. Model interpretation through lower-dimensional posterior summarization. *arXiv:1905.07103 [stat]* (2020).
