# Supplementary material for: A Synergistic Mindsets Intervention Protects Adolescents from Social Stress
Source: Res Sq. 2021 May 28:rs.3.rs-551170. Preprint. [Version 1] doi: 10.21203/rs.3.rs-551170/v1 (PMC8168396; doi:10.21203/rs.3.rs-551170/v1)
Supplement: Supplement 2 [file 90ddfc5d714f0ead2cab24b9.pdf]

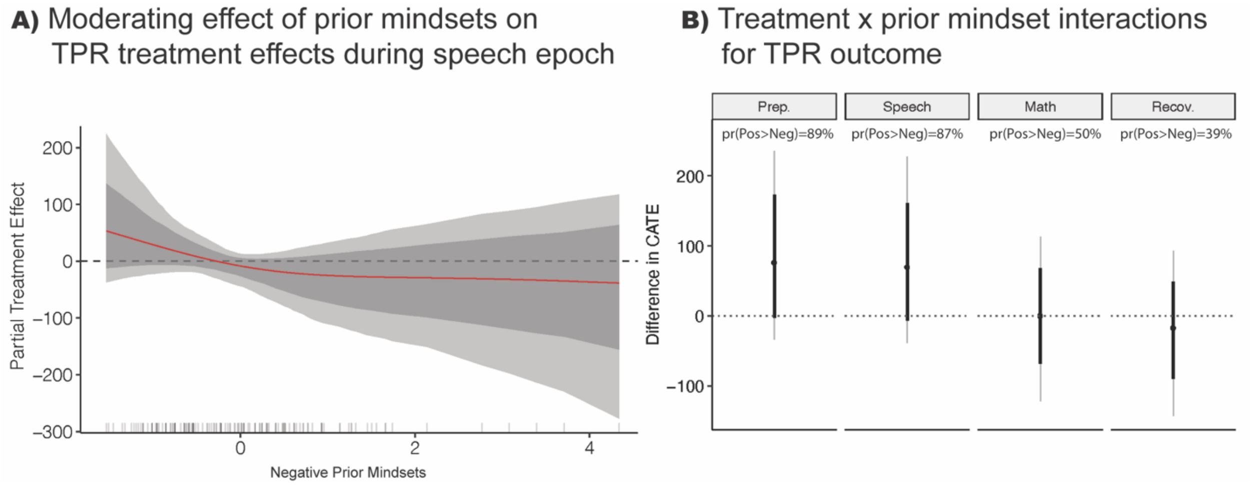

Extended Data Fig. 1. ***In Study 3 (N = 160), prior mindsets moderated the treatment effect on TPR during stressful TSST epochs. In (A), an additive summary of the posterior distribution of treatment effects, by negative prior mindset levels, in (B) and the interaction between treatment and prior mindsets on TPR responses across TSST epochs.*** Note: TPR = total peripheral resistance (in dyne-sec x cm^5). In (A) the y-axis represents the "partial" treatment effect, which corresponds to the offset from the average treatment effect, holding other potential moderators constant.

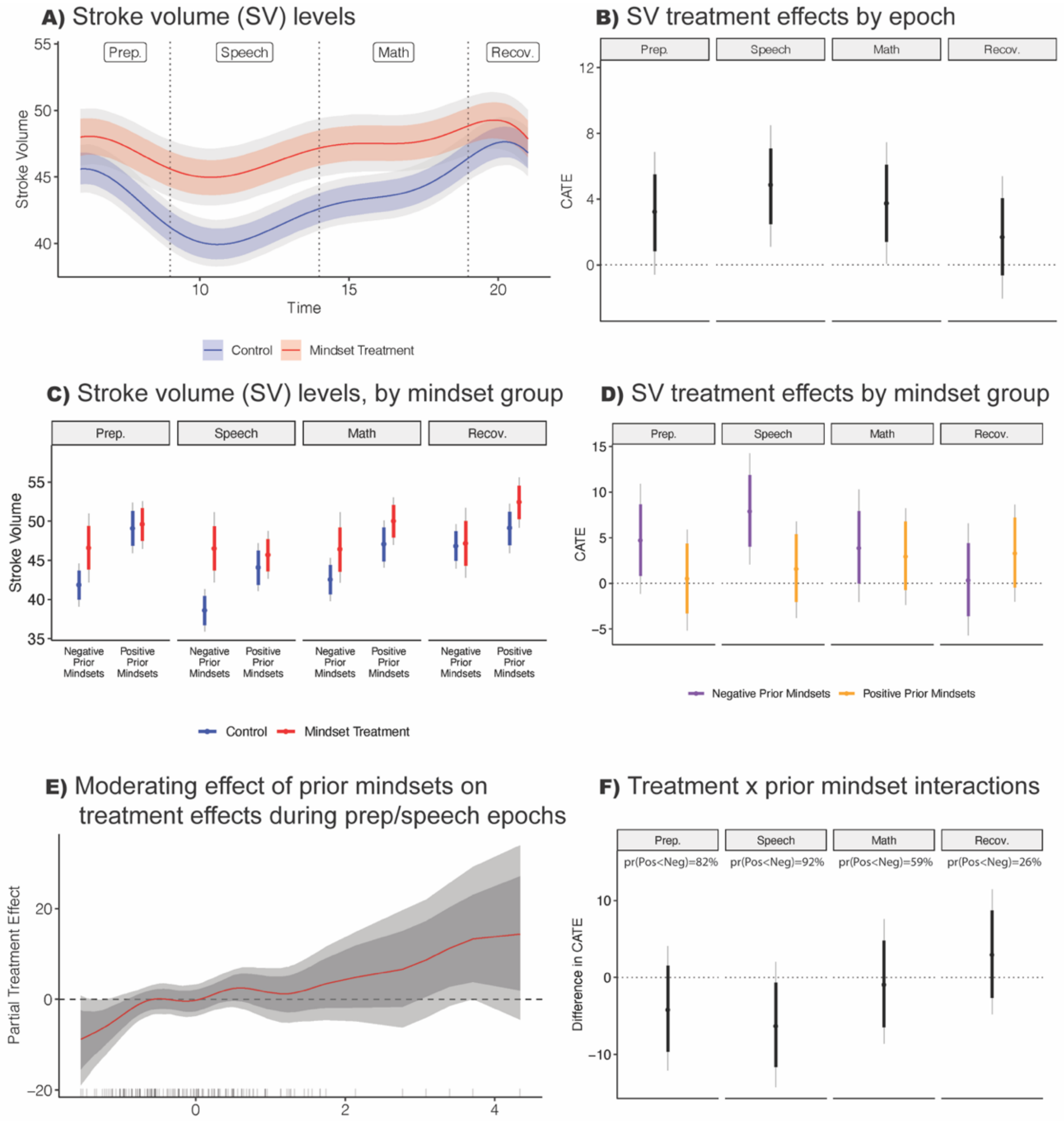

Extended Data Fig. 2. ***In Study 3 (N = 160), the synergistic mindsets intervention improved cardiovascular responses to the Trier Social Stress Test (TSST) as indexed by Stroke Volume (SV) in milliliters (A, B), especially for participants with negative prior mindsets during the most stressful epochs (C, D). Prior mindsets moderated the treatment effect on SV during stressful TSST epochs (E), and the interaction between treatment and prior mindsets on SV responses across TSST epochs (F).*** Effects of the intervention on stroke volume (SV)—the amount of blood ejected from the heart during each beat, in *ml*—were tested because challenge (relative to threat) responses increase SV to facilitate actively addressing stressors [42,43,45]. Thus, we anticipated those experiencing challenge-type stress during the stressful TSST epochs should exhibit relatively higher stroke volumes as their bodies distribute oxygenated blood to optimize performance, whereas threatened individuals were expected to have lower stroke volumes during stressful epochs of the TSST as their bodies seek to concentrate blood in the core. Consistent with predictions, there was a substantial positive treatment effect during the speech and math epochs: ATE for Prep = 3.24 ml [0.832, 5.501], Speech = 4.87 ml [2.473, 7.067], Math = 3.751 [1.409, 6.078], Recovery = 1.69 [-0.624, 4.056]. The effect was 4.2x greater among participants with negative prior mindsets relative to positive mindsets. In (C) and (D), the prior mindset subgroups used to display the different treatment effects were generated by implementing a hands-off Bayesian decision-making algorithm that maximized the

differences among the mindset groups in terms of the outcome, without using information on the magnitudes of the treatment effects.

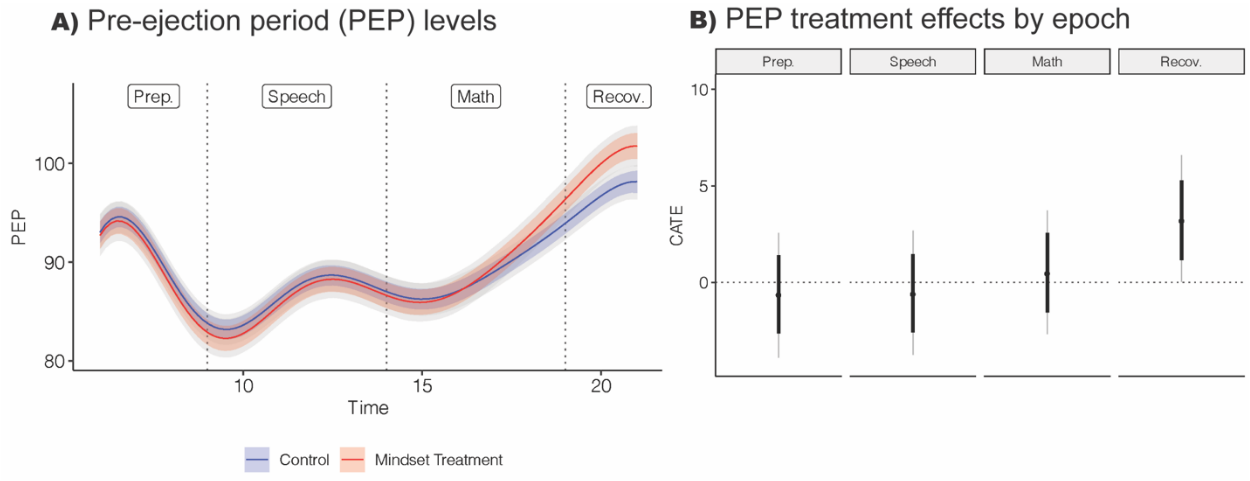

Extended Data Fig. 3. ***In Study 3 (N = 160), the effect of the synergistic mindsets intervention on pre-ejection period (PEP) in milliseconds across Trier Social Stress Test (TSST) epochs (A, B).*** Pre-ejection period (PEP)—which assesses the contractile force of the heart by measuring the time from onset of ventricular depolarization to aortic valve opening—was examined to test for effects of the intervention on sympathetic arousal. Threat type responses are associated with sustained vigilance for sources of harm and prolonged stress responses relative to challenge type responses [81]. Thus, challenge is associated with more a rapid recovery to homeostasis after stress offset. Whereas all participants should show PEP decreases (leading to a more rapid heart rate) relative to baseline during the stressful epochs [45,81] (see Fig. 1), condition differences may emerge during the recovery period, because controls should be slower to return to homeostasis relative to treated individuals. Indeed, a positive treatment effect of 3.18 *ms* [1.159, 5.286] was found during the recovery epoch only, as expected [45].

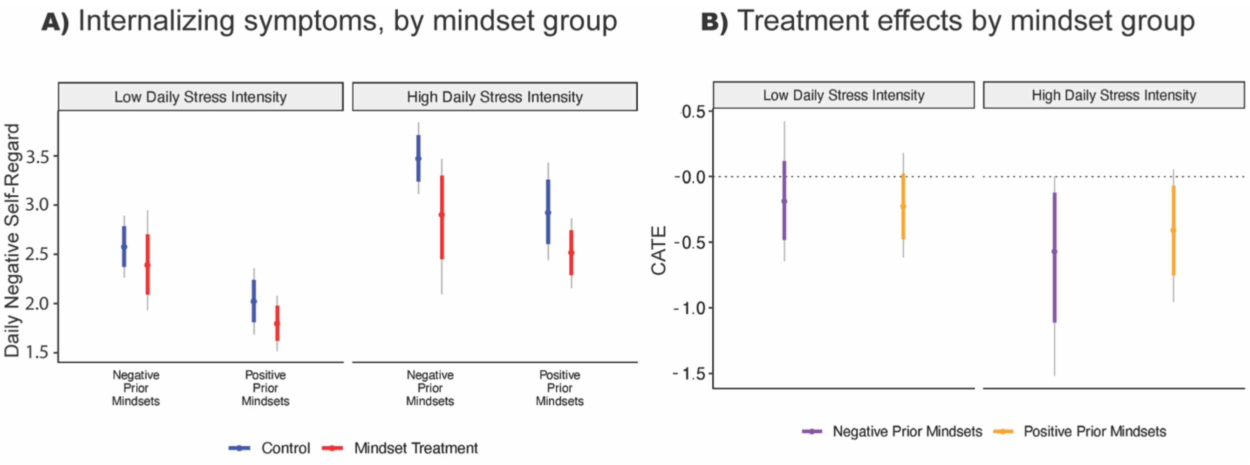

Extended Data Fig. 4. ***In Study 4 (N = 118, n <=*** **1213** ***observations), the synergistic mindsets intervention reduced internalizing symptoms the most among people with negative prior mindsets, on their most highly stressful days (A,B).*** Note: The CATEs (or conditional average treatment effects) are: Low Daily Stress Intensity, Negative Prior Mindsets CATE = -.186 [-.484, .118], Positive Prior Mindsets CATE = -.225 [-.479, .0215]; High Daily Stress Intensity, Negative Prior Mindsets CATE = -.571 [-1.113, -.122], Positive Prior Mindsets CATE = -.-.406 [-.753, -.070]. Hence, the CATE was 40% for negative prior mindsets participants on high stress days relative to positive prior mindsets participants. The prior mindset subgroups used to display different treatment effects were generated by implementing a hands-off Bayesian decision-making algorithm that maximized the differences among the mindset groups in terms of the outcome, without using information on the magnitudes of the treatment effects.

Outcome	Correlation of outcome with negative prior mindsets in the control condition, r =	*df* =
Study 1		
Event appraisals	**.26**	1388
Response appraisals	**.16**	1382
Study 2		
Quiz#1 appraisals	Not assessed	NA
Quiz#2 appraisals	Not assessed	NA
Study 3		
Total peripheral resistance (TPR) (active, non-baseline epochs)	**.20**	1699
Study 4		
Daily internalizing symptoms	**.27**	523
Study 5		
Anxiety	**.38**	170

Extended Data Table 1. Negative prior mindsets predicted outcomes in the control condition in each of the five experiments. Negative prior mindsets are a multiplicative term of event- and response-focused mindset measures assessed prior to the intervention. For Study 3, the active epochs were all epochs except the baseline (pre-stressor) epochs.

Outcome<br>Predictor	**Treatment effect *β* =**	*se*=	*t* =	*df* =	***P* =**
Study 1					
Negative mindsets	**-0.280**	0.036	7.743	2716	**<.001**
Event appraisals	**0.117**	0.036	3.309	2601	**<.001**
Response appraisals	**0.200**	0.039	5.099	2592	**<.001**
Study 2					
Negative mindsets	**-0.465**	0.076	6.119	696	**<.001**
Quiz#1 appraisals	**0.410**	0.070	5.893	755	**<.001**
Quiz#2 appraisals	**0.183**	0.073	2.515	720	**.006**
Study 3					
Negative mindsets	**-0.563**	0.129	4.368	157	**<.001**
TPR reactivity					
Treatment main effect	**-0.569**	0.131	4.330	151 (2291 obs)	**<.001**
Treatment x Negative prior mindsets	**-0.205**	0.108	1.894	151 (2291 obs)	**.029**
Study 4					
Negative mindsets	**-0.413**	0.173	2.389	111	**.009**
Internalizing symptoms					
Treatment main effect	**-0.219**	0.125	1.763	118 (1045 obs)	**.039**
Treatment x Daily stress intensity	**-0.113**	0.053	2.131	118 (1045 obs)	**.017**
Treatment x Daily stress intensity x Negative prior mindsets	**-0.080**	0.056	1.421	118 (1045 obs)	**.077**
Salivary cortisol					
Treatment main effect	**-0.282**	0.094	2.986	115 (1213 obs)	**.001**
Study 5					
Negative mindsets	**-0.483**	0.076	6.378	347	**<.001**
Anxiety					
Treatment main effect	**-0.192**	0.121	1.587	332	**.057**
Treatment x Negative prior mindsets	**-0.191**	0.087	2.197	332	**.014**

Extended Data Table 2. Treatment effect estimation with traditional linear regression analysis and classical null hypothesis testing reproduces the primary findings from each of the five studies. β = magnitude of the treatment effect on a z-scored outcome variable. *P* = one-tailed p-value, due to directional hypotheses. TPR = total peripheral resistance, a measure of threat-type stress responding. All outcome models controlled for the same set of pre-random-assignment variables (when measured): prior stress mindsets, prior fixed mindsets, prior global stress (the perceived stress scale), prior self-esteem, and female gender. The Study 3 TPR models and the Study 4 internalizing and cortisol models were estimated using linear mixed effects modeling with a random intercept for each

participant because of the repeated measures; the remaining models were typical linear regressions. The negative prior mindsets variable is the multiplicative term of prior stress mindsets and prior fixed mindsets; in every case the models also included the lower-order interactions.
