# Supplementary material for: A Synergistic Mindsets Intervention Protects Adolescents from Social Stress
Source: Res Sq. 2021 May 28:rs.3.rs-551170. Preprint. [Version 1] doi: 10.21203/rs.3.rs-551170/v1 (PMC8168396; doi:10.21203/rs.3.rs-551170/v1)
Supplement: Supplement 3 [file 0f5b80a7f3a4cfa3d4e02cfe.pdf]

Stress intervention

0:54 / 0:56

Your brain and body: How they work for you

We are developing a new program for students next year. We would like to invite you to help improve it. This program shares scientific facts about the human brain and body during challenging periods of your life, like the start of a new year in school. For example, did you know that:

- Experiences of difficulty, struggle, and frustration are actually important ways for your brain to learn and grow stronger;
- Sometimes people feel anxious or "stressed" when they are learning new, hard things in school, but that's okay--it means your body is preparing to support your learning.
- People who know these facts--that the brain changes with learning, and that the body mobilizes resources to support your learning--are better prepared for the difficulties they face in school.

If we can learn from your experiences, we can teach these powerful ideas to students like you more effectively in the future.

0:50 / 0:50

Why Did We Make This Program?

We are scientists at Stanford University, the University of Texas, the University of Chicago, and the University of Rochester. We made this program because many students say they're excited about starting a new school year, but they're stressed too, because there are a lot of challenges. Students take new classes, have new teachers, and meet new people. They may also think about what they want to do in life, and who they want to be.

This program can help future students to develop powerful new skills in school, rather than having stress overwhelm them. This makes them prepared to accomplish the things they want in life and helps them to contribute to the world around them.

But the program needs more real-life examples from students at your school. After all, we're not the experts in what it's like to be in your school. You are.

0:54 / 0:54

Before we continue, please share an experience from your own life. That way we can present better examples in the program next year.

On your own piece of paper, please answer this question:

Think of a time when you were working something important for school and you felt stressed or anxious

because you were worried you wouldn't be able to do a good job. Please choose a time when your body had a strong stress reaction. This could mean your heart was pounding or you might have started to sweat or shake or feel out of breath.

Try to choose something you care about, because we will come back to this example later. This might be a time when you had to give a presentation in front of people, take an important test, or complete a difficult assignment.

On your own paper, please explain what thoughts you were having or what your body was doing that made you realize you were stressed (write 2 to 5 sentences).

0:10 / 0:10

Let's get started

Thank you for your input!

Next, please read more about how the brain learns in the face of challenges, and how this relates to your time in school.

0:35 / 0:35

The new secrets of success

Have you ever noticed that the way people talk about success and achievement can make it seem like it just comes naturally to some people?

This is a problem because it makes it seem like success and achievement only come from some “natural ability” that a person is either born with or not. Scientists know this isn’t true.

The problem with believing that achievement is supposed to come easily is that it can make people miss an important insight about how you can achieve the things that matter to you: **Experiences of struggle, frustration, and difficulty can be your friend.**

0:43 / 0:43

Neuroscientists now understand how this works

Neuroscientists (the people who study how learning happens in the brain) know that when you're faced with difficult challenges and you keep trying until you get better, your brain grows new connections and becomes better at taking on new challenges in the future.

As you do this more and more, things that used to be hard start to feel easier. And, when something does feel really difficult, your brain learns how to respond more effectively to that challenge.

It's a lot like the way rigorous exercise makes your muscles really sore at first but, with training, your muscles don't just get stronger, they also recover more quickly when you do push them to their limit.

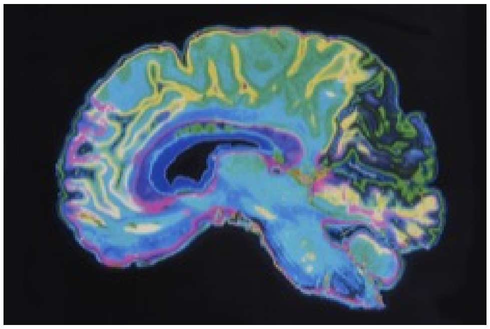

Source: Nature Reviews Neuroscience, 2012

0:26 / 0:26

The brain is like a muscle: When you use it, it gets stronger (and smarter)

Why do scientists say that the harder you work, the smarter your brain becomes? As you may know, your brain has billions of tiny cells, called neurons.

A neuron has a cell body, a long branch (an axon), and tiny branches (dendrites). It's the dendrites that connect the neuron to other neurons.

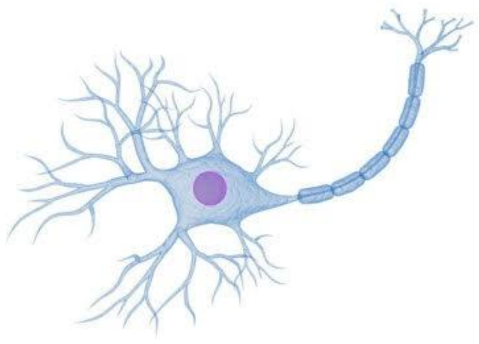

0:31 / 0:31

Stronger connections make a stronger brain

The connections between neurons can be weak or strong. When you work hard to learn something new—like a new type of math problem or a difficult skill like presenting in front of people—the connections in your brain get stronger.

The more time you spend learning hard things and practicing them, the stronger those connections get. Over time, these stronger connections can make you smarter in a subject, so things that used to feel hard start to feel easier.

0:38 / 0:38

Doing challenging things strengthens your brain's connections

When people don't know this information about how the brain works, they tend to avoid frustration and difficulty by not doing things that feel hard.

But by avoiding difficulty, they're robbing themselves of their best opportunities to develop skills and strengths that will help them achieve the things that are important to them.

Although stressful experiences feel unpleasant in the moment, they are the path through which everyone who ever became really good at something got to where they are. They are the way your neurons grow new, stronger connections—connections that help your brain get smarter.

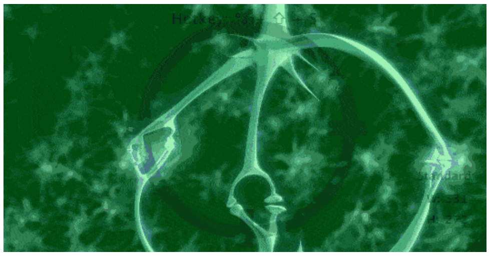

Neurons in your brain grow connections when you learn from challenges.

0:37 / 0:37

Expanding your limits

The point isn't that you should constantly be pushing yourself to improve. Rest is important too, and only you know how much challenge and difficulty is right for you.

Instead, the point is that you are not limited by what you can

do right now. You have the power to embrace challenges so you can grow your skills.

Difficulty, struggle, and frustration when you're learning something are not signs that you've reached your limits, they're signs that you're expanding your limits.

That means you can learn to use the difficulty and stress of your hardest class assignments to build yourself up.

0:53 / 1:08

Let's hear from a scientist.

It's not always obvious how the difficult assignments we do in school can make our brains stronger. Some people might think that truly "smart" people always have an easy time. But that's not true.

Let's hear from Daniel Greene, a scientist at Stanford University. Here's what he says:

"One of the most amazing facts about human learning is that you're making your brain stronger even when you don't realize it, as long as you're working hard to teach yourself

something new. When people do hard work in school—like public speaking or math problems—it can feel stressful. And if they don't understand that stress, they think it means, 'Oh no, I don't belong here.' But in fact, that stress is an indicator that your understanding is deepening. It's not a sign that you're ***not*** *learning. It's a sign that you* ***are*** *learning."*

0:29 / 0:29

The surprising science of the human stress system

Why does Dr. Green say this? Psychophysiologists (the scientists who study the relationship between the body and the mind) now know that stress and anxiety can be a powerful tool.

That feeling when your heart is pounding and your palms are sweating is your body's stress response—it's your body kicking into high gear so you can do the hard work necessary to help your brain grow and strengthen connections between neurons.

0:53 / 0:54

The body's stress response

When we struggle to accomplish something we care about, whether it's a difficult math problem, a challenging athletic or musical skill, or an important personal goal, we get anxious. We think of that as a bad thing—a feeling that gets in the way when we're trying to perform.

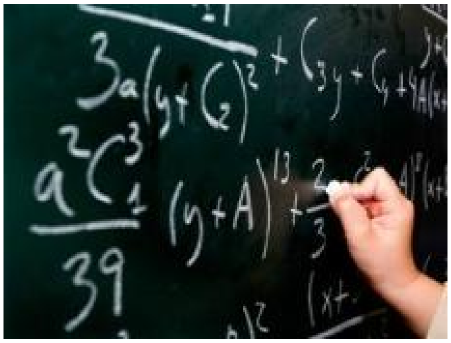

But the truth is that stress response is exactly what your brain needs to take on difficult challenges and learn from them.

Think about it. Millions of years ago our ancestors had to fight off danger or hunt down prey so they could eat. They needed their body's stress responses to give them a performance boost, to keep their families alive. Today, our challenges are different but still important. Our body's stress responses help our minds stay alert so we can rise to the challenge and do well in math or science or any other class that makes us anxious.

0:46 / 0:46

How does this work?

Oxygen is the fuel for our brain. When your heart starts beating faster, it's delivering an extra liter of oxygenated blood to your muscles and brain each minute. This means your brain has more fuel to help you think.

Pumping all that extra blood to your brain and muscles means you need to breathe faster to keep that blood rich with oxygen. That's why you breathe more heavily when you're stressed.

Your muscles and brain both generate extra heat when they work harder, so you begin sweating to cool your body down. Sweating allows you to keep performing at that higher level without overheating.

When your body detects a challenge, it also releases catecholamines (like adrenaline) that help you think more quickly and solve problems better.

0:29 / 0:29

Your body is preparing you

People often mistake their body's stress response for a sign that they're in a situation they can't handle. It's easy to do: racing heart, fast breathing, and sweating are also ways our bodies respond in emergencies, when we're in real trouble.

But thinking your stress response is bad is a mistake that can actually cause you to perform worse. **If you think your stress response is a problem, you're more likely to be worried about it and get distracted from performing your best.**

0:31 / 0:31

Using your body's stress response to energize your performance

You are now armed with the key information you need to use your body's stress response effectively next time you feel it kicking in while you're trying to perform or master something difficult. **When you start to feel anxious, try to remind yourself that this is your body's way of helping you to rise and meet the challenge you're facing.**

That should help you to spend less time worrying about the fact that you feel anxious so you can focus on what you're doing and let your body's stress response give you the extra boost you need.

0:31 / 0:31

How could *you* use your body's stress response effectively?

Think back on the stressful school situation you wrote about at the beginning of this program--one where you wanted to perform at your best, but felt stressed about it. Or, if you want

to, think of a new example of a time you felt anxious and stressed about an assignment or test for school.

We’re going to share three ways that science shows you can use the information you learned today to help you in the future. (Please get your own paper ready.)

0:47 / 0:56

How do you use your body's stress responses?

Below are three ways that people don’t just survive but **thrive** during stressful situations like the one you wrote about. Which are ones you have used before?

Please click *all* of the ones you’ve tried. Then, on your own paper, please tell us what it would look like for you, personally, to do them again in the future. If you haven't tried them, then you can tell us how someone could get started using these tips.

- ☐ Remind yourself that the feelings of confusion and struggle when doing difficult school work won't last forever because, as you face and work through those struggles, your brain is growing smarter

☐ Get the support (or develop the skills) that you need to be able to master your challenges

☐ Remember that your body's stress response (heart beating faster, breathing more heavily etc.) is there to help you reach your peak performance level when you're facing a difficult challenge

0:38 / 0:38

You selected:

"Remind yourself that the feelings of confusion and struggle when doing difficult school work won't last forever because, as you face and work through those struggles, your brain is growing smarter."

On your own piece of paper, answer this question: **How could you use this kind of strategy again in an upcoming stressful situation like the one you wrote about?** For instance, you could mention:

- Visualizing your brain's neurons growing stronger connections while you are in the midst of struggling on an assignment
- Think back on times when you used to not know something (maybe when you were a child) but, over time, came to learn more about it and get better at it

0:28 / 0:28

You selected:

"Get the support (or develop the skills) that you need to be able to master your challenges."

On your own piece of paper, answer this question: **How could you use this kind of strategy again in an upcoming stressful situation like the one you wrote about?** For instance, you could mention:

- Leveling up your skills, especially in your areas of weakness, by getting help from teachers or friends or trying to study in a new way
- Spend time with people who care about you

0:41 / 0:41

You selected:

"Remember that your body's stress response (heart beating faster, breathing more heavily etc.) is there to help you reach your peak performance level when you're facing a difficult

challenge."

On your own piece of paper, answer this question: **How could you use this kind of strategy again in an upcoming stressful situation like the one you wrote about?** For instance, you could mention:

- If you find yourself feeling anxious, remind yourself that your body's stress response is there to help you
- Remind yourself that your body’s stress response will only get in your way if you spend energy worrying about it
- If you are feeling stressed about an assignment, you could use your increased alertness to help you stay focused on the task

0:43 / 0:43

You said you haven't tried this yet:

"Remind yourself that the feelings of confusion and struggle when doing difficult school work won't last forever because, as you face and work through those struggles, your brain is growing smarter."

We’d like to hear from you about the ways you might begin to

put this into practice. On your own piece of paper, answer this question: **What are some initial steps you could imagine yourself taking to do this during an upcoming stressful situation like the one you wrote about?** For instance, you could mention:

- Visualizing your brain's neurons growing stronger connections while you are in the midst of struggling on an assignment
- Think back on times when you used to not know something (maybe when you were a child) but, over time, came to learn more about it and get better at it

0:34 / 0:34

You said you haven't tried this yet:

"Get the support (or develop the skills) that you need to be able to master your challenges."

We'd like to hear from you about the ways you might begin to put this into practice. On your own piece of paper, answer this question: **What are some initial steps you could imagine yourself taking to do this during an upcoming stressful**

situation like the one you wrote about? For instance, you could mention:

- Leveling up your skills, especially in your areas of weakness, by getting help from teachers or friends or trying to study in a new way
- Spend time with people who care about you

0:18 / 0:47

You said you haven't tried this yet:

"Remember that your body's stress response (heart beating faster, breathing more heavily etc.) is there to help you reach your peak performance level when you're facing a difficult challenge."

We'd like to hear from you about the ways you might begin to put this into practice. On your own piece of paper, answer this question: **What are some initial steps you could imagine yourself taking to do this during an upcoming stressful situation like the one you wrote about?** For instance, you could mention:

- If you find yourself feeling anxious, remind yourself that your body's stress response is there to help you

- Remind yourself that your body’s stress response will only get in your way if you spend energy worrying about it
- If you are feeling stressed about an assignment, you could use your increased alertness to help you stay focused on the task

0:09 / 0:09

What do other students say?

On the next two pages, hear more about these ideas from past students. Then we will need your help one last time.

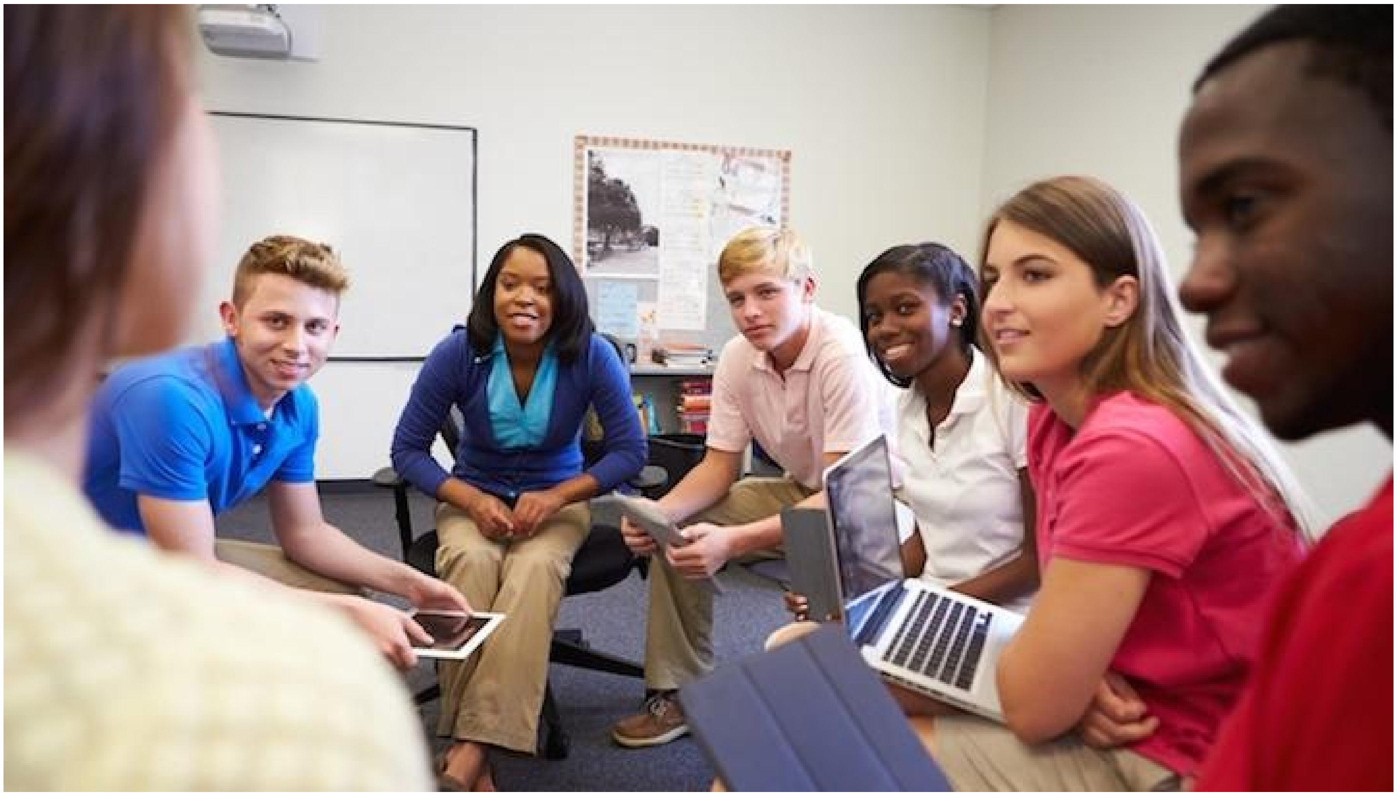

0:40 / 0:40

J.H., a senior in college:

"People always tell me to relax and not stress about tests or big projects. But that never felt like good advice because I actually care a lot about what I'm doing and I feel motivated to do a good job. I'm glad someone finally took the time to explain that it's a good thing that I'm not taking the easy road. ***It makes sense that telling yourself 'don't stress' doesn't work*** *when you've chosen to do something hard but important. Instead,* ***I can use my body's stress response to do better at the things that matter a lot to me****. I feel way more comfortable knowing that my body and brain are working to*

help me even when I don't know it--and that I can harness that power when I need to."

0:18 / 0:37

Help us explain this to other students

Science shows that no matter who you are—whether you've earned straight Fs or straight As—you can grow your intelligence, and your body's stress system is there to support you.

Sadly, some students don't know this. They may think, "I'm already smart, so I don't have to work hard" or "when I feel stressed, it means I'm going to fail."

This puts them at a disadvantage. They miss out on the chance to grow their abilities. This could keep them from becoming the person they want to be and from doing something good for themselves, their community, or the world.

0:53 / 1:36

Help us explain this to other students

Here is where we really need your input. Please turn to a new piece of paper.

Think about new students coming to your school next year. Imagine a student who is struggling in class and is feeling discouraged. Maybe they feel like the stress is piling up so high that they can't do anything about it. And so they're having trouble getting motivated.

On your own piece of paper, please write a personal message to a student who will be coming to your school next year.

1. First, **describe a time when you found your schoolwork challenging or stressful.** Write 2 to 5 sentences.
2. Second, what could you say to help a new student next year who might be experiencing something similar to understand that **these challenging and stressful experiences can improve with time, because your brain can learn to deal with them?** On your paper, write 1-2 paragraphs.

For instance, you can mention:

- That the brain forms new, stronger connections when it learns from challenges, or
- That the body's stress response system--your heart rate, your breathing, and so on--supports the brain's learning by fueling it with the oxygen it needs to take on hard challenges

We may choose some of your statements to share with students (anonymously) during stressful times next year. *Remember, the more powerful your message is, the more helpful it will be to next year's student.* You don't have to worry about spelling or grammar – just focus on sharing your thoughts. If you want, you can sign your letter.

0:38 / 0:38

One final point: Not all stressful experiences are good

Thank you for your response.

Here's a final point. The lessons of this program apply to normal experiences in school, like stress we feel when we are

trying to learn or master a difficult concept or skill. That's "good stress," and you should trust that your body's responses are helping you perform.

But sometimes people experience trauma—stress that is outside their control and harmful. If you experience trauma, you could reach out to a parent, teacher, counselor, or other adult who you trust for advice.

Powered by Qualtrics
